# Advanced dwarf mongoose optimization for solving CEC 2011 and CEC 2017 benchmark problems

**DOI:** 10.1371/journal.pone.0275346

**Published:** 2022-11-02

**Authors:** Jeffrey O. Agushaka, Olatunji Akinola, Absalom E. Ezugwu, Olaide N. Oyelade, Apu K. Saha

**Affiliations:** 1 School of Mathematics, Statistics, and Computer Science, University of KwaZulu-Natal, Pietermaritzburg, KwaZulu-Natal, South Africa; 2 Department of Computer Science, Federal University of Lafia, Lafia, Nigeria; 3 Department of Computer Science, Faculty of Physical Sciences, Ahmadu Bello University, Zaria, Nigeria; 4 Department of Mathematics, National Institute of Technology Agartala, Tripura, India; UCSI University, MALAYSIA

## Abstract

This paper proposes an improvement to the dwarf mongoose optimization (DMO) algorithm called the advanced dwarf mongoose optimization (ADMO) algorithm. The improvement goal is to solve the low convergence rate limitation of the DMO. This situation arises when the initial solutions are close to the optimal global solution; the subsequent value of the alpha must be small for the DMO to converge towards a better solution. The proposed improvement incorporates other social behavior of the dwarf mongoose, namely, the predation and mound protection and the reproductive and group splitting behavior to enhance the exploration and exploitation ability of the DMO. The ADMO also modifies the lifestyle of the alpha and subordinate group and the foraging and seminomadic behavior of the DMO. The proposed ADMO was used to solve the congress on evolutionary computation (CEC) 2011 and 2017 benchmark functions, consisting of 30 classical and hybrid composite problems and 22 real-world optimization problems. The performance of the ADMO, using different performance metrics and statistical analysis, is compared with the DMO and seven other existing algorithms. In most cases, the results show that solutions achieved by the ADMO are better than the solution obtained by the existing algorithms.

## 1. Introduction

Optimization occurs naturally in many human endeavors, and most human decisions go through an optimal process. Optimization is deeply rooted in many branches of science, for example, a radiation reactor system with minimal emission in physics, maximizing profit in businesses, survival of the fittest in ecology, and production line design in a manufacturing system that satisfies a set of constraints [1]. There are two established methods of solving optimization problems: the mathematical and metaheuristic approach. Each method comes with specific drawbacks; for instance, the mathematical methods are gradient-dependent, which implies that the initial starting position of the population plays a significant role in its performance [2]. The drawbacks of the two methods, coupled with the fact that global optimization problems are complex in nature, and the ease of mimicking nature’s way of solving problems, have significantly contributed to the surge in the rate at which researchers are proposing nature-inspired algorithms [3].

Many aspects of nature have been a source of inspiration for developing metaheuristic algorithms. Over the years, many optimization researchers have successfully used different natural phenomena as a source of inspiration to develop metaheuristic algorithms [2]. For instance, the genetic algorithm’s (GA) source of inspiration is natural selection in the theory of evolution [4]. The intelligent way birds flock together inspired the design of the particle swarm optimization (PSO) [5]. Generally, problems in various domains ranging from the traveling salesman problem [6], optimal control [7]and many more, have been solved using nature-inspired metaheuristic algorithms. The research community believes the success of nature-inspired metaheuristic algorithms is attributed to imitating the best ways nature solves problems.

Some authors have criticized the over-reliance on metaphor-based paradigm by the nature-inspired metaheuristic algorithms [8]; however, there is consensus on the many successes recorded by these algorithms in finding solutions to complex benchmark optimization problems [9]and real-world problems in the engineering domain [10]. Like all real-world optimization problems, almost all engineering problems come with several nonlinear and complex constraints depending on the design criteria and safety rules. The optimization process of all nature-inspired metaheuristic algorithms consists of steps that mimic the problem-solving process of the natural phenomena they mimic.

No one algorithm exists that solves all optimization problems optimally, meaning each can only solve some problems optimally and others suboptimally. Hence the argument for developing a new or improved high-performance algorithm that solves specific problems. Also, many novel metaheuristic algorithm developers have cited the no-free lunch theory as a basis for regularly developing new algorithms, claiming that the proposed algorithms find better solutions for optimization problems. There is also the claim by the newly proposed algorithms of balancing exploration and exploitation to better search the problem space [11]. The claim by some metaheuristic algorithms of drawing inspiration from nature is debatable, and so is the claim of novelty and strong optimization capability.

A list of some newly proposed metaheuristic algorithms is presented in Table 1. Interested readers are referred to [3, 12–15]for a more detailed list of metaheuristic algorithms proposed within the past five decades. Also, a detailed survey of metaheuristic algorithms that outlined their components and concepts, intending to analyze their similarities and differences, is given in [16, 17]. Interestingly, some of the inspirations claimed in the articles are drawn from human inventions rather than naturally occurring phenomena. For instance, the social network search (SNS) draws inspiration from the social network user’s efforts to gain more popularity, a human invention rather than a naturally occurring phenomenon.

**Table 1:** Some nature-inspired metaheuristic algorithms with their source of inspiration (2019-2021).

Algorithm	Inspiration	Reference
Group teaching optimization algorithm	Group teaching mechanism	[23]
Black widow optimization algorithm	unique mating behavior of black widow spiders.	[24]
Chaos Game Optimization	some principles of chaos theory	[25]
Adolescent Identity Search Algorithm (AISA)	process of identity development/search of adolescents	[26]
Atomic orbital search	basic principles of quantum mechanics	[27]
A novel metaheuristic optimizer inspired by behavior of jellyfish in the ocean	behavior of jellyfish in the ocean	[28]
Quantum dolphin swarm algorithm	dolphin swarm algorithm	[29]
Arithmetic optimization algorithm	Arithmetic operators	[30]
Advanced arithmetic optimization algorithm	Advanced arithmetic operators	[31]
Ebola Optimization Search Algorithm (EOSA)	Ebola virus	[32, 33]
Golden ratio optimization method (GROM)	Growth in nature using the golden ratio of Fibonacci series	[34]
Bald eagle search optimization algorithm	bald eagle	[35]
Black Hole Mechanics Optimization	mechanics of black holes	[36]
Capuchin search algorithm	capuchin monkeys	[37]
Tiki-taka algorithm	football playing style	[38]
Cooperation search algorithm	team cooperation behaviors in modern enterprise	[39]
Aquila Optimizer	Aquila bird	[40]
The Sailfish Optimizer	The Sailfish group hunting	[41]
Social Network Search	social network user’s efforts to gain more popularity	[42]
Sine–cosine and Spotted Hyena-based Chimp Optimization Algorithm (SSC)	a hybrid algorithm is developed which is based on the sine–cosine functions and attacking strategy of Spotted Hyena Optimizer (SHO)	[43]
Archimedes optimization algorithm	law of physics Archimedes’ Principle	[44]
Battle royale optimization algorithm	a genre of digital games knowns as “battle royale.”	[45]
Thermal Exchange Metaheuristic Optimization Algorithm	Newton’s law of cooling	[46]
African vultures optimization algorithm	African vultures	[47]
The Red Colobuses Monkey	Red Colobuses Monkey	[48]
Remora optimization algorithm	parasitic behavior of remora	[49]
Red deer algorithm (RDA)	Red deer	[50]
Pelican optimization algorithm	Pelican	[51]
Reptile optimization algorithm	Hunting crocodiles	[52]
Squirrel search algorithm	Squirrels	[53]
Dwarf mongoose optimization	Dwarf mongoose	[54]
Human Felicity Algorithm	Quest for the Evolution of Human Society	[55]
Giraffe kicking optimization	Giraffe	[56]
Competitive search	Competition	[57]
Criminal search optimization algorithm	Police strategies	[58]
Horse herding optimization algorithm	Horse herd	[59]
Gaining‑sharing knowledge based algorithm	Gaining and sharing knowledge during the human life span	[60]

Researchers have also hybridized existing metaheuristic algorithms instead of developing an entirely new metaheuristic algorithm. So many works of literature exist that have hybridized one metaheuristic algorithm with another. Some examples include the firefly algorithm hybridized with chaos theory [18], the hybridization of ant colony strategy and harmony search scheme (HPSACO) [19], particle swarm optimizer hybridized with a variant of cuckoo search called the island-based cuckoo search, and highly disruptive polynomial mutation (iCSPM) [20], hybridization of self-assembly and particle swarm optimization (SAPSO) [21], fuzzy controllers hybridized with slime mound algorithm (SMAF) [22].

Further to the novel research outcomes resulting from the metaheuristic method and their associated hybrid or variant algorithms, the area of applicability presents more research prospects in the field. Optimization problems in engineering and machine learning are currently being researched, with the former having received considerable research efforts. Machine learning, specifically deep learning, has demonstrated interesting performances in image analysis [61–64]but still suffers from architectural composition resulting from combinatorial problems, which require an optimization process as a solution. Efforts to address these using heuristic methods such as in [65, 66], have further revealed the complexity of the optimization problem. To remedy this, studies [32, 67–70]have approached the use of metaheuristic algorithms, or a hybrid of metaheuristic algorithms, or even some high-performing variants. In [32], the authors applied a metaheuristic algorithm to support the selection of an optimal combination of convolutional neural network hyperparameters (CNN) to address classification problems in digital mammography and chest x-ray.

Similarly, metaheuristic algorithms were employed to address the challenge of network weight optimization in [70]. Authors in [69]have also adapted metaheuristic algorithms to the evolution of neural architectures, a combinatorial problem consisting of finding the best neural network components for obtaining the best performing architecture suitable for solving a particular classification problem. In [68], the problem of feature selection for reducing classifier bottleneck was addressed using the GA metaheuristic method. The study of [67]investigated the performance of a chaotic-theory-enabled FA metaheuristic in improving the dropout regularization of deep learning models. Several other studies have investigated the use of hybrids of metaheuristic algorithms in solving object detection, segmentation, classification, and image generation and reconstruction problems. However, with new variants and high-performing hybrids of these algorithms still being researched, it further reveals that improving performance in handling optimization problems in machine learning is opening up new research frontiers. As a result, the motivation for deepening the optimization process of existing optimization algorithms through designing variants and hybrids is furthering research in metaheuristic algorithms.

Although the dwarf mongoose optimization (DMO) algorithm [54]is inspired by the foraging and social-behavioral structure of the dwarf mongoose, modeling the unique compensatory behavioral adaptations of the dwarf mongoose in DMO has led to a limitation of slow convergence due to the role the value of the alpha female plays in the updating process. Therefore, in this study, an improvement on the DMO is presented that mimics four (4) different aspects of the life of the dwarf mongoose, eliminating the limitation posed by the value of the alpha. The four social structural adaptations are modeled for the optimization process: the alpha and subordinate group, the foraging and seminomadic behavior, the predation and mound protection, and the reproductive and group splitting behavior. The study identified some major stages of activities observed in the group, namely predation, territory circuiting, reproduction, group splitting, and foraging. These processes are repeated until termination criteria are met. The proposed improved algorithm is used to solve CEC 2011 and 2017 benchmark functions, consisting of 30 classical and hybrid composite problems and 22 real-world optimization problems.

Considering the dwarf mongoose has been the source of inspiration for DMO and all the natural phenomena explaining their existence and survival, this presents a promising and improved optimization process. The research question now is: considering the competitive performance demonstrated by the DMO [54], which models only a selected phenomenon in the natural phenomenon of the dwarf mongoose, could a better and improved optimization process and performance be achieved by modeling all fundamental and existential phenomena in nature? Motivated by this research question, a detailed study of literature on dwarf mongooses was examined, and all fundamental concepts were extracted for consideration. Interestingly, critical processes and stages of the dwarf mongoose were found, which motivated the optimization process and mathematical models resulting in the proposed advanced dwarf mongoose optimization (ADMO) algorithm presented in this study. The following are the technical contributions of this study:

- A new optimization process model is designed with four stages: predation, foraging and semi-nomadism, reproduction, and group splitting.
- Mathematical models were developed to model each of the four stages described in (i).
- The optimization process design in (i) and the models in (ii) were applied to design a new variant of the DMO algorithm, namely the ADMO.
- Exhaustive experimentation was carried out using CEC 2017 and CEC 2011 constraint benchmark optimization functions for comparative analysis of ADMO against the base algorithm and other methods.

The rest of the paper is organized as follows: In Section 2, the dwarf mongoose optimization algorithm (DMO) is presented. Section 3 presents the advanced dwarf mongoose optimization algorithm (ADMO). The experimental setup, results, and detailed discussion are presented in Section 4. Finally, the conclusion and future work is presented in Section 5.

## 2. The dwarf mongoose optimization algorithm

This section presents an overview of the DMO, including its inspiration and the optimization processes. Also, this section is divided into two subsections to enable the smooth presentation of the various aspect of the DMO. The source of inspiration and the basic behavior of the dwarf mongoose used for the DMO are discussed in subsection one. In contrast, the implementation of the model is discussed in subsection two.

### 2.1. Inspiration

The DMO drew its inspiration from the dwarf mongoose, also called *Helogale*. They are found in areas with abundant termite mounds, rocks, and hollow trees used for hiding and protection. Africa’s semidesert and savannah bush are typical habitats of dwarf mongoose. They are the smallest known African carnivore and live in a family group that is a matriarchy [71, 72]. The social order of the mongoose family is such that the females and the young are ranked higher than the males and the juveniles, respectively. The division of labor and altruism within these groups is the highest that has been recorded for a mammal, and each mongoose serves as a guard, babysitter, attacking predators, or attacking conspecific intruders [73–76].

The dwarf mongoose has developed specific behavior and adaptations to survive in its natural habitat. These adaptions and behavior relate to predation avoidance and nutrition. They are not known to have a killer bite but rather a skull-crushing bite using the prey’s eye for orientation. Also, no cooperative killing of large prey has been observed in the dwarf mongoose family. These adaptations restrict their prey’s size and significantly affect the mongooses’ social behavior and ecological adaptations to achieve individual and family nutrition [76]. The DMO is modeled after two compensatory behavioral adaptations of the mongoose, namely

- Prey size, space utilization, and group size
- Food Provisioning.

### 2.2. The DMO model

**Fig 1:**
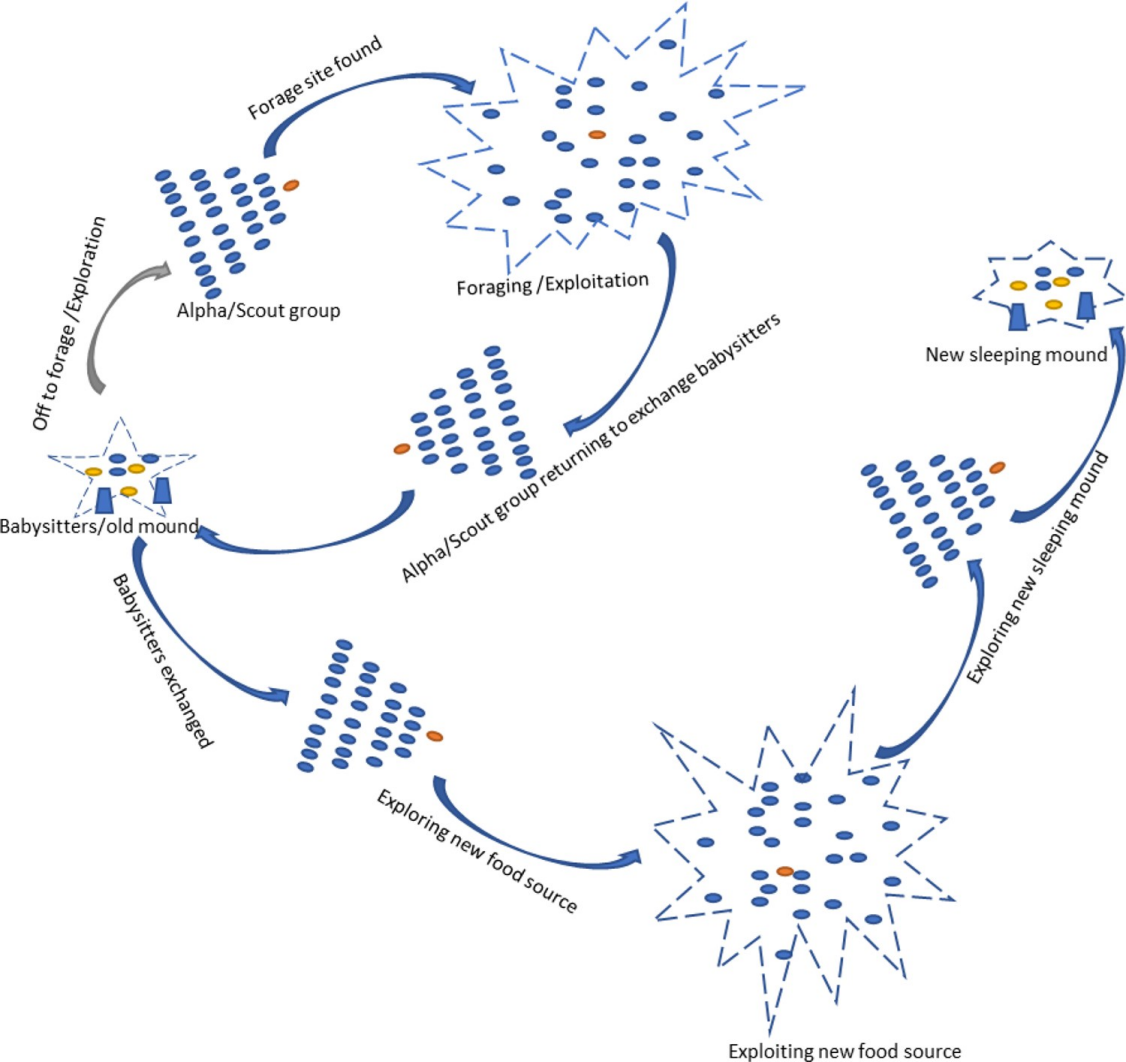
The optimization procedures of the DMO.

The DMO [54]algorithm simulates the compensatory adaptation of the dwarf mongoose as the forage. The dwarf mongoose population is divided into the alpha group, scouts, and babysitters. Each group contributes to the compensatory behavioral adaptation, which leads to a seminomadic way of life in a territory (problem space) large enough to support the entire group. The scouting for new mounds and foraging are done simultaneously by the same group of mongooses in DMO. The optimization procedures of the proposed DMO algorithm are represented in three phases, as shown in Fig 1. The red dot signifies the alpha leading the family (blue dots) to find a food source, leaving behind the babysitters with the young (exploration). Once the food source is found, the entire group feeds extensively in the area (exploitation). The family returns intermittently to exchange babysitters and repeats the cycle.

The DMO starts by randomly initializing the candidate population and computing the fitness of each. The selection of alpha female (*α*) is based on Eq 1.


$$\alpha =\frac{{fit}_{i}}{\sum_{i=1}^{n}{fit}_{i}}$$ (1)

To update a candidate’s food position, the DMO uses the expression given in Eq 2.

$$X_{i+1}=X_{i}+phi*peep$$ (2)

where *phi* is a uniformly distributed random number [–1,1], the *peep* is assumed to be the alpha female’s vocalization that helps keeps the family bound together on the same path. The sleeping mound (*sm*) is updated after every iteration using Eq 3.


$${sm}_{i}=\frac{{fit}_{i+1}-{fit}_{i}}{max\{|{fit}_{i+1},{fit}_{i}|\}}$$ (3)

The average value of the sleeping mound *sm* is computed by Eq 4.


$$\varphi =\frac{\sum_{i=1}^{n}{sm}_{i}}{n}$$ (4)

The scout group is simulated using Eq 5. The scouts must look for the new sleeping mound because the dwarf mongooses are seminomadic and never return to the previous sleeping mound. This behavior activates the exploration, and DMO models the scouting and foraging to be carried out simultaneously [76].

$$X_{i+1}=\left\{ \begin{array}{l} X_{i}-CF*phi*rand*{[X}_{i}-\vec{M}]\;if\;\varphi_{i+1}>\varphi_{i}\\ X_{i}+CF*phi*rand*{[X}_{i}-\vec{M}]\;else \end{array}\right.$$ (5)

where,

*rand* is a random number between [0,1],

$CF={\left(1-\frac{iter}{Max_{iter}}\right)}^{\left(2\frac{iter}{Max_{iter}}\right)}$ is the collective-volitive movement control parameter and $\vec{M}=\sum_{i=1}^{n}\frac{X_{i}\times {sm}_{i}}{X_{i}}$ determines the movement of the mongoose to the new sleeping mound.

The pseudocode for the algorithm is given in algorithm listing 1 (Fig 9, S1 File).

## 3. Advanced dwarf mongoose optimization algorithm model

**Fig 2:**
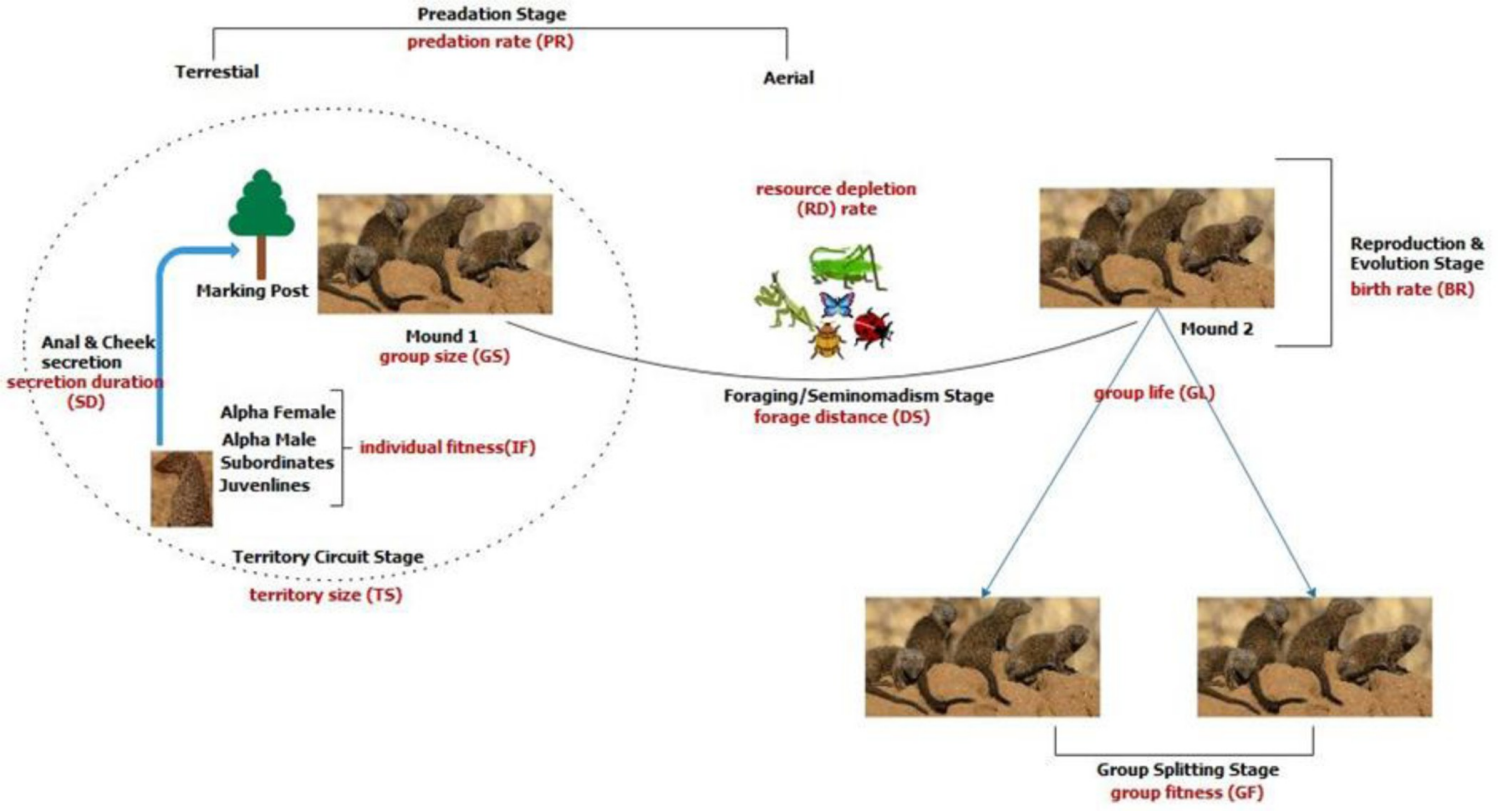
The model of the proposed optimization process for ADMO.

The section presents the advanced dwarf mongoose optimization algorithm (ADMO). The ADMO is proposed to solve the low convergence rate limitation of the DMO. This situation arises when the initial solutions are close to the optimal global solution; the subsequent value of the alpha must be small for the DMO to converge towards a better solution. The proposed improvement incorporates other social behavior of the dwarf mongoose, namely, the predation and mound protection and the reproductive and group splitting behavior to enhance the exploration and exploitation ability of the DMO. The ADMO also modifies the lifestyle of the alpha and subordinate group and the foraging and seminomadic behavior of the DMO. The optimization procedures of the proposed ADMO algorithm are represented in three phases, as shown in Fig 2. This model shows five (major) stages in the dwarf mongoose mounds. These stages are territory circuit, predation, foraging, reproduction, and group splitting.

The search space of the proposed algorithm is a population of dwarf mongoose individuals initialized using Eq 6. Search for the news areas in the search space is achieved using the exploration mechanism of the algorithm. The criterion leading to the exploration phase’s optimization process is conditioned on comparing foraging distance covered and territory size values. When the foraging distance exceeds the given territory size, the algorithm transits to the exploration phase; otherwise, the intensification phase is maintained. Obtaining the best solution depends on a sustained high rate of avoiding predators. Predation often weakens the quality of individuals in the search space. At the same time, avoidance of predation and increased foraging outside a territory space produces high-quality individuals in the search space.

### 3.1. Population initialization

The ADMO population is initialized with candidate dwarf mongooses (X), as shown in Eq (6). The population is generated stochastically between the given problem’s upper bound (U) and lower bound (L).


$$X=\{x_{1},x_{2},x_{3},\ldots .x_{n}\}$$ (6)

Where *n* is the population size for an arbitrary dwarf mongoose mound and each *x*_*i*_ is initializezd using Eq (7) and the position of all individuals in *X* in the mound is represented by (8).

$$x_{i}=rand\times (U-L)$$ (7)

$$dmp(X)=P=[\begin{array}{lllll} x_{1,1} & x_{1,2} & \cdots & x_{1,d-1} & x_{1,d}\\ x_{2,1} & x_{2,2} & \cdots & x_{2,d-1} & x_{2,d}\\ \vdots & \vdots & x_{i,j} & \vdots & \vdots \\ x_{n,1} & x_{n,2} & \cdots & x_{n,d-1} & x_{n,d} \end{array}]$$ (8)

where *x*_*i*,*j*_ denotes the position of the j^th^ dimension of the i^th^ population, n denotes the population size, and d is the dimension of the problem computed using *dmp*(*X*).

### 3.2. Alpha and subordinate groups

Once the population is initialized, gender-based compositional differences (M1 and M2) for male and female alpha members and alpha vector ($\vec{AV}$) for representing alpha characteristics is applied to obtain the alpha male (*x*_*alpham*_) and female (*x*_*alphaf*_). The best individual, say *x*_*best*_, in the population is used for benchmarking members of the alpha group. So that if we randomly select *x*_*i*_ and *x*_*j*_ from the population to represent male and female respectively and mutate them to *x*_*best*_, then Eqs (9) and (10) hold for the alpha male and alpha female.

$$x_{alpham}=x_{i}+(rand(0,1)\times M1\times \vec{AV})$$ (9)

$$x_{alphaf}=x_{j}+(rand(0,1)\times M2\times \vec{AV})$$ (10)

where M1 and M2 represents (1+*rand*(0,1)) and (0.5+*rand*(0, 1)) (0.5+*rand*(0, 1)) and $\vec{AV}$ is computed using $\vec{AV}=\frac{x_{best}}{2}$. We now have n-2 individuals to partition among the subordinate and juvenile groups. The subordinate often represents the largest set of individuals in a mound, followed by the juvenile. The size of individuals in subordinate set *S* and juvenile set *J* is computed using $s=floor(\frac{n-2}{3})$ and $j=floor(\frac{n-2}{4})$ respectively. Their members are allocated by sorting *X* using their individual fitness values, and the first *s* are allocated to *S* and *j* allocated to *J*.

### 3.3. Foraging and semi-nomadism stage

The foraging and seminomadic nature of dwarf mongooses are motivated by the fact that food sources are scattered, requiring an extensive search by the individual to find sufficient food for itself. This foraging act often takes an intensive search over a long distance (*fd*), in Eq (12), which will most times be greater than territory size (*ts*) in Eq (13). The *x*_*alphaf*_ is known to lead the foraging party, hence its position *dmp*(*x*_*alphaf*_) helps to compute *fd*≥*ts*. Cessation of foraging is aided by predation rate *pr* and birth rate *br*, thereby lowering energy output due to reduced energy input from nutrition and, in that case, *fd*<*ts*. This is summarized in Eq (11) which computes the new state of any individual *x*_*i*_ in the group. Reduced space utilization leads to depleted food sources hence reduced individual fitness. In addition, the lower the group size (*gs*), the higher *fd*, while *ts* is computed using the summation of the age (in this case the *age* function expressed in Eq 13) of anal and cheek marking of all individuals in the group.


$$x_{i}=\left\{ \begin{array}{l} x_{i}+rand(0,1)*{[x}_{best}-x_{alphaf}]\;fd\geq ts\\ x_{i}+rand(-1,1)*{[x}_{best}-x_{alphaf}]\;otherwise \end{array}\right.$$ (11)

$$fd=dmp(x_{alphaf})*(pr+br)$$ (12)

$$ts=\sum_{i=1}^{n}age(x_{i})$$ (13)

Where *pr* and *br* represent the average predation and birth rates for a mound. The position of all individuals in the group is updated after every iteration using *dmp*(*X*)+1 for each *x*_*i*_.

### 3.4. Predation and mound protection

The dwarf mongoose population suffers from terrestrial and aerial attacks, wading off the attack using a group approach. The terrestrial attack is categorized into attacks from another group of dwarf mongooses and attacks from other animals. When another group of dwarf mongoose attacks *φ*_1_, *x*_*alpham*_ is credited with leading all fights, followed by the subordinates $s=floor(\frac{n-2}{3})$, juvenile $j=floor(\frac{n-2}{4})$, and *x*_*alphaf*_. When animals that are not dwarf mongooses attacks *φ*_2_ the mound, only the subordinates $s=floor(\frac{n-2}{3})$ and juvenile $j=floor(\frac{n-2}{4})$ attack the enemy. Fatalities are often associated with the more aggressive juvenile group, thereby depleting their number in the mound group. Group fitness *gf* in Eq (14) and density of marking post *mp* in Eq (15) determines if the predator wins the group or loses to the group in the case of *φ*_1_ attack while only *gf* determines their win in the *φ*_2_ attack.

$$gf=\sum_{i=1}^{n}fit(x_{i})$$ (14)

$$mp=\frac{\sum_{i=1}^{n}age(x_{i})}{n}$$ (15)

where *fit* represents the fitness value of the individual *x*_*i*_.

We simulate the case of *φ*_1_, *φ*_2_, or neither of (*φ*_1_ and *φ*_2_) in every iteration, with the impact and update on the loss of a group member shown in Eq (18). We represent the loss effect using a tuple of current group members, group fitness, and the density of marking posts. We simplify Eq (18) by showing how cases 1 and 2 are computed using Eqs (16) and (17).

$$X_{\varphi_{1}},{gf}_{\varphi_{1}},{mp}_{\varphi_{1}}=(X-(x_{a},x_{a+1}\ldots x_{l})),\sum_{i=1}^{n-l}fit(x_{i}),\frac{\sum_{i=1}^{n-l}age(x_{i})}{n}$$ (16)

$$X_{\varphi_{2}},{gf}_{\varphi_{2}},{mp}_{\varphi_{2}}=(X-(x_{b},x_{b+1}\ldots x_{k})),\sum_{i=1}^{n-k}fit(x_{i}),\frac{\sum_{i=1}^{n-k}age(x_{i})}{n}$$ (17)

$$X,gf,mp=\left\{ \begin{array}{c} {(X}_{\varphi_{1}},{gf}_{\varphi_{1}},{mp}_{\varphi_{1}})\;attack\;is\;\varphi_{1}\\ (X_{\varphi_{2}},{gf}_{\varphi_{2}},{mp}_{\varphi_{2}})\;attack\;is\;\varphi_{2}\\ \left(X,gf,mp)\;not(\varphi_{1}\;\mathrm{and}\;\varphi_{2}\right) \end{array}\right.$$ (18)

where *a*, *b*, *k*, and *l* denote the index of the first subordinate in the population, the index of the first juvenile in the population, the number of subordinates, and the number of juveniles, respectively, affected during an attack. Note that *k* must satisfy $0\leq k\geq s\;where\;s=floor(\frac{n-2}{3})$, and $0\leq l\geq j\;where\;j=floor(\frac{n-2}{4})$.

### 3.5. Reproduction and group splitting

The *x*_*alphaf*_ is the only female who can raise young in a mound, rendering female subordinates and female juveniles incapable of childbearing. All cases (100%) of estruses cycle among the *x*_*alphaf*_ leads to pregnancy, while only 62.5% of estruses cycle for subordinates leads to pregnancy. However, the young resulting from the female subordinates are either killed at birth or unable to survive since they cannot suckle. As a result, an increase in group size *gs* is strictly the exclusive right of *x*_*alphaf*_. Studies showed that the average frequency of young by the subordinate female is 0.66 compared with 9.66 for the alpha female. As a result, the reproduction (addition) of young into the population is updated using Eq (19).

$$X=(X+(x_{1},x_{2}\ldots x_{alphayoung}))$$ (19)

where *alphayoung* is computed thus: $alphayoung=floor(\frac{n*9.66}{100})$.

For group splitting, dwarf mongooses are contractors rather than expansionists to preserve an economically defensible area to avoid depletion of resources (e.g., food) for the group and promote reproduction. Although group splitting is not frequent, when it does occur, the splinter group, often motivated and led by independent females, exits the mound for the main group and moves to another territory to form a new group. This often decreases *gs* and *gf*. Because this group exit often excludes the *x*_*alpham*_ and *x*_*alphaf*_ The subordinate (S) members often constitute the independent female and her followers breaking away from the main group. We simulate the impact of the group splitting on group size using Eq (20.


$$X=\left\{ \begin{array}{l} X-\{x_{i}|x_{i}\in S⋀2\leq i\geq rand(s)\}\;rand(1,3)==2\\ \;X\;otherwise \end{array}\right.$$ (20)

Individual fitness depends on the *cost* and *benefit* relationship the individual partakes in the group. Notably, the fitness value of dominant members is higher than those of the subordinates; hence two cost factors and benefit factors: $\vec{CST1},\vec{BF1}$ and $\vec{CST2},\vec{BF2}$ for the dominant group and subordinates, respectively. Meanwhile, since individual fitness sums up the group fitness, we compute the fitness and secretion (anal and cheek) of an arbitrary individual subordinate as follows in Eqs (21) and (22):

$$fit(x_{i})=(x_{i}*\vec{CST2}-\;rand(0,1*\vec{BF2})$$ (21)

$$age(x_{i})=(x_{i}*\vec{CST2})$$ (22)

The values for the vector pair $\vec{CST1},\vec{BF1}$ are obtained by duplicating the best and worst individuals among the dominant group and dividing both by the size of the dominant group. Similarly, the values for the pair $\vec{CST2},\vec{BF2}$ are computed in the same manner except that the best and worst individuals are selected from the subordinate groups, and the division operation is done using the size of the subordinate group.

### 3.6. ADMO procedure

To achieve the algorithmic design of the proposed ADMO model, we first present the procedural description of the model. This is to illustrate the flow of processes in the algorithm and flowchart. The optimization strategy obtained from the dwarf mongoose begins with population initialization. This is followed by some major activities observed in the group. These activities include predation, territory circuiting, reproduction, group splitting, and foraging. These processes are repeated until termination criteria are met. A representation of the pseudocode for the algorithm is given below

- Generate a defined number of dwarf mongoose individuals.
- Each dwarf mongoose belonging to each subgroup is evaluated using a domain-specific fitness function to obtain the current best individual. The current best is explicitly defined as the global best.
- Based on the fitness evaluation of all individuals, sort the population and assign individuals to the subgroups: alpha male, alpha female, subordinates, and juveniles
- Initialize and set domain-specific control parameters such as Group fitness (*gf*), the density of marking post (*mp*),
- For a defined number of iterations, and while the termination condition is not satisfied, REPEAT
  - Compute using Eqs (12) and (13) the model on the territory circuit stage
  - Compute using Eq (11) the model on the foraging phase to obtain foraging distance (fd) and territory size (ts)

If foraging distance exceeds settlement territory size, THEN

    - Mongoose foraging due to depletion in food in settlement space

Otherwise,

    - Mongoose still have food to sustain the group in the current settlement (mounds)
- Derive the nature of predation by computing the values for *φ*_1_, *φ*_2_, or neither of (*φ*_1_ and *φ*_2_)

If *φ*_1_, or *φ*_2_ holds, THEN

Check if its

    - Compute using the first condition on Eq (18)

Otherwise

- Compute using the second condition on Eq (18)

otherwise

    - Compute using the third condition on Eq (18)
- Generate a random number of young alpha species and add them to the population: reproduction/evolution phase

- Using Eq (20), split the group to achieve two new dwarf mongoose groups existing independently
- Compute the current best fitness and update the global best
- Go up to check if the termination condition is not satisfied. Otherwise, move to the next line
- RETURN best solution

**Fig 3:**
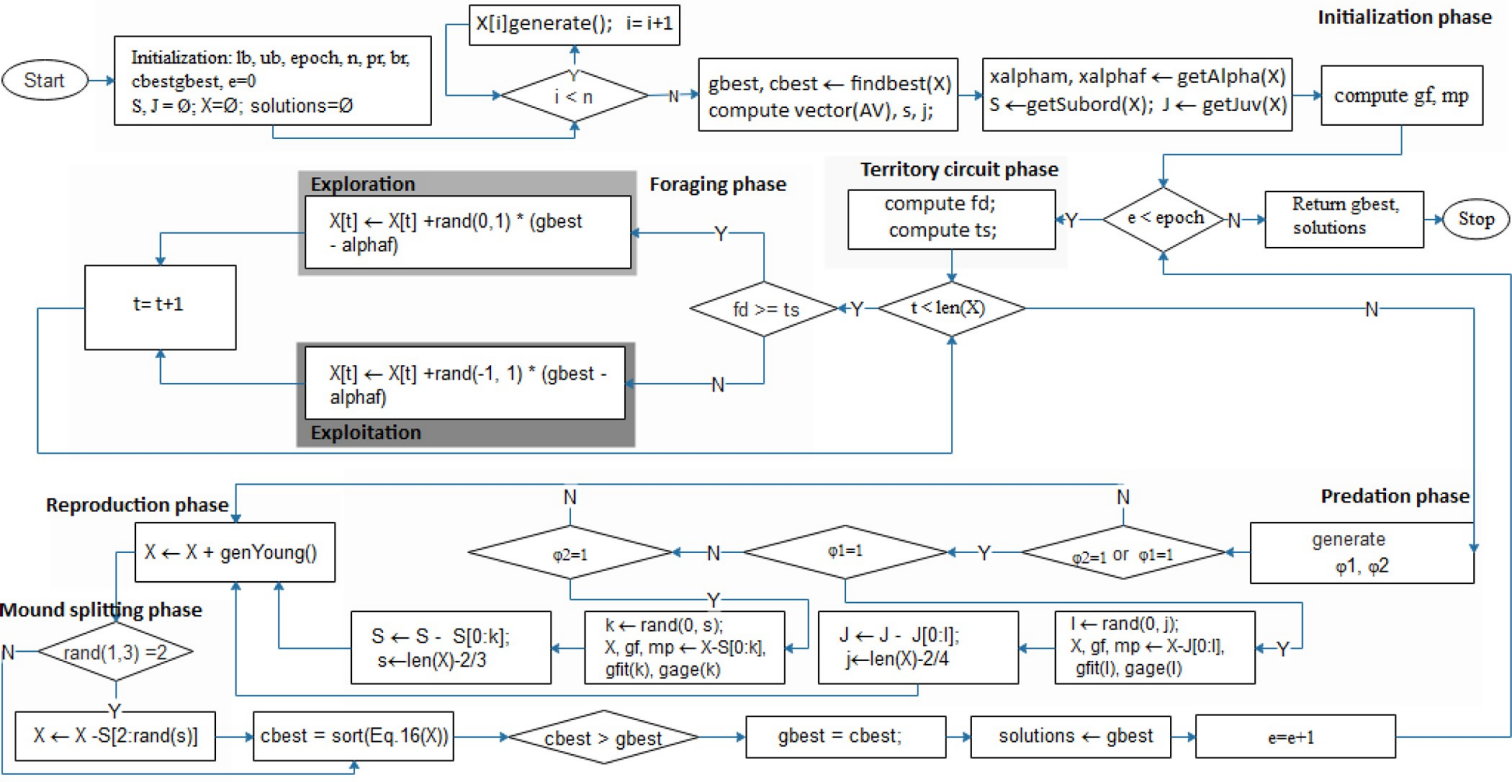
The flowchart of the proposed ADMO.

In Fig 3, a detailed procedure representation is described, with all identified model stages highlighted. In addition, we indicate where the exploration and exploitation phases of the proposed ADMO are balanced.

### 3.7. Computational complexity

The computational complexity of the DMO and eight (8) other algorithms are measured as defined in [77], and their results are presented in Tables 2–4. The algorithms are implemented using MATLAB R2020b, Windows 10 OS environment, Intel Core i7-7700@3.60GHz CPU, and 16G RAM. The time (T0) needed to run the program (D=10, 30, 50) below is measured:

x=0.55;

Fori=1:1000000

x=x+x;

x=x/2;

x=x*x;

x=sqrt(x);

x=log(x);

x=exp(x);

x=x/(x+2);

end

**Table 2:** The computational complexity for D=10.

Algorithm	T0	T1	T2	(T2-T1)/T0
ADMO	0.0372	0.5989	1.8312	33.12634
LSHADEcnEpSin			8.9691	225.0054
LSHADE			5.1503	122.3495
DMO			1.9943	37.51075
LSHADE_SPACMA			2.1835	42.59677
UMOEA			9.9169	250.4839
WOA			10.0548	254.1909
AOA			19.8781	518.2581
CPSOGSA			9.9769	252.0968

**Table 3:** Computational complexity for D=30.

Algorithm	T0	T1	T2	(T2-T1)/T0
ADMO	0.0372	0.7234	2.2312	40.53226
LSHADEcnEpSin			9.9691	248.5403
LSHADE			6.1593	146.1263
DMO			2.5743	53.10215
LSHADE_SPACMA			3.6724	79.27419
UMOEA			11.1169	279.3952
WOA			12.9548	328.8011
AOA			39.8789	1052.567
CPSOGSA			11.9764	302.5

**Table 4:** Computational complexity for D=50.

Algorithm	T0	T1	T2	(T2-T1)/T0
ADMO	0.0372	1.0076	2.9071	51.06183
LSHADEcnEpSin			10.9691	278.7688
LSHADE			7.1503	176.1129
DMO			2.9943	64.39247
LSHADE_SPACMA			7.7564	181.4194
UMOEA			13.9969	360.1613
WOA			16.7548	434.2984
AOA			49.8781	1324.71
CPSOGSA			12.9769	332.7419

In the same vein, the time (T1) needed to run *f18* (D=10, 30, 50) from the CEC 2017 test suit 200,000 times, and the mean time (T2) for five (5) runs of the same function is measured. The value of (*T*2− *T*1)*/T*0 gives the complexity of the respective algorithm. From the results in Tables 2–4, the ADMO returned the minimum values compared to the other eight (8) algorithms. Conclusively, the computational complexity of the ADMO is relatively low and easy to implement.

### 3.8. Conceptual advantage of the ADMO

The performance of the proposed ADMO in finding the global optimum solutions to different optimization problems can be theoretically attributed to the following:

- The ADMO stochastically creates a set of candidate solutions for given optimization problems and improves these solutions using the enhanced exploratory and exploitation ability of DMO. The enhancement results from the group splitting, antipredation, and reproduction activities of the dwarf mongoose, which further mutates the candidate solutions.
- The problem search space is explored and exploited as the dwarf mongooses forage across the territory. In ADMO, the foraging depends on comparing the foraging distance and territory size, ensuring the ADMO escapes local optima.
- The ADMO also has only one parameter that can be tuned.

As listed in Algorithm 2 (Fig 10, S1 File), the following algorithm reflects the mathematical model and procedural listing for the ADMO model.

The search space of the proposed algorithm is a population of dwarf mongoose individuals initialized using Eq (6) and Lines 2-4 of Algorithm 2 (Fig 10, S1 File). Search for the news areas in the search space is achieved using the exploration mechanism of the algorithm. The criterion leading to the optimization process entering the exploration phase is conditioned on comparing the values of foraging distance covered and territory size. The algorithm transits to the exploration phase when the foraging distance exceeds the given territory size. Otherwise, the intensification phase is maintained. Obtaining the best solution depends on a sustained high rate of avoiding predators. Predation often weakens the quality of individuals in the search space. In contrast, avoiding predation and increased foraging outside a territory produces high-quality individuals in the search space.

## 4. Results and discussion

The proposed improvements of the ADMO were tested to establish performance using CEC 2011 and 2017 benchmark functions, consisting of 30 classical and hybrid composite problems and 22 real-world optimization problems. The results of ADMO for benchmark functions were compared with that of DMO and seven existing population-based metaheuristic algorithms, namely: arithmetic optimization algorithm (AOA), constriction coefficient based (PSO) and GSA (CPSOGSA), whale optimization algorithm (WOA), linear population size reduction success-history based adaptive DE (LSHADE), and covariance matrix learning with Euclidean neighborhood ensemble sinusoidal LSHADE (LSHADE-cnEpSin), LSHADE with semi-parameter adaptation hybrid with CMA-ES (LSHADESPACMA) and united multi-operator EA (UMOEA). The algorithms are carefully selected because of their track records and performance in different CEC competitions. Also, they represent different metaheuristic categories available in the literature. All the algorithms and optimization problems considered were implemented using MATLAB R2020b, and Table 5 presents the different algorithm control parameters used for the experiments. Notably, the control parameters given in Table 5 are as used in their original references. Windows 10 OS environment, Intel Core i7-7700@3.60GHz CPU, and 16G RAM were used to conduct the experiments. The results of 51 and 25 independent runs of each algorithm for CEC 2017 and CEC 2011, respectively, are collated using the “Best, Worst, Average, and SD” performance indicators. Further statistical analysis was carried out using mean, standard deviation, Friedman test, and Wilcoxon signed test.

**Table 5:** Algorithm control parameters.

Algorithm	Ref	Name of the parameter	Value of the parameter
**AOA**	[30]	*α*	5
		*μ*	0.05
**LSHADEcnEpSin**	[78]	freq_inti	0.5
		pb	0.4
		ps	0.5
**CPSOGSA**	[79]	<p1, <f>2	2.05
**LSHADE**	[80]	p_best_rate	0.11
		memory_size	5
		arc_rate	1.4
**LSHADE_SPACMA**	[81]	L_Rate	0.8
		Pbest, Memory size (H), and Arc_rate	Same as LSHADE
		The threshold for SPA activated	max_nfes/2
		Probability Variable (*FCP*)	0.5
**UMOEA**	[82]	Par.MinPopSize	4
		Par.prob_ls	0.1
		PS2	4+floor(3*log(Par.n))
		PS1	Par.PopSize
**WOA**	[83]	a	2-t*((2)/Max_iter)
		C	2*r2
		A	2*a*r1-a

### 4.1. CEC 2017 Benchmark test function

The results of all the algorithms used in this study are presented in this section. In addition to the performance metrics stated earlier, this study also presented the solution error measure defined as *f*(*x*)−*f*(x*). The solution error gives the difference between the best result (*x*) found in one run of the algorithm and the globally known result *f*(x*) for a specific benchmark function.

#### 4.1.1. Results of ADMO for CEC 2017

The results obtained by ADMO across the four (4) different dimensions (D=10, 30, 50, and 100) are presented in Tables 6–9. Clearly, the performance of ADMO across the different dimensions is competitive. Specifically, from Table 6, the ADMO found the global optimal result for 27 benchmark functions (D=10) at least once and consistently found the optimal solution for 12 out of the 27 functions over 51 runs. It can be seen from Table 7 (D=30) that the ADMO successfully found the solution for 3 benchmark functions, 2 of which were consistent over 51 runs and 1 at least once. Looking at Table 8, the ADMO found optimal solutions for 3 benchmark functions at least once and was not consistent over the 51 runs.

**Table 6:** Results for ADMO in 10 dimensions.

Function	Best	Worst	Mean	Std
**F1**	0.00E+00	0.00E+00	0.00E+00	0.00E+00
**F3**	0.00E+00	0.00E+00	0.00E+00	0.00E+00
**F4**	0.00E+00	0.00E+00	0.00E+00	0.00E+00
**F5**	0.00E+00	1.99E+00	4.80E-01	6.22E-01
**F6**	0.00E+00	0.00E+00	0.00E+00	5.77E-10
**F7**	1.00E+00	1.27E+01	9.78E+00	3.40E+00
**F8**	0.00E+00	1.99E+00	2.00E-01	4.80E-01
**F9**	0.00E+00	0.00E+00	0.00E+00	0.00E+00
**F10**	3.00E-01	1.29E+02	2.64E+01	3.44E+01
**F11**	0.00E+00	0.00E+00	0.00E+00	0.00E+00
**F12**	0.00E+00	0.00E+00	0.00E+00	1.03E-05
**F13**	0.00E+00	2.00E-01	0.00E+00	3.34E-02
**F14**	0.00E+00	0.00E+00	0.00E+00	0.00E+00
**F15**	0.00E+00	0.00E+00	0.00E+00	9.99E-04
**F16**	0.00E+00	3.00E-01	1.00E-01	9.50E-02
**F17**	0.00E+00	1.40E+00	5.00E-01	4.50E-01
**F18**	0.00E+00	0.00E+00	0.00E+00	1.06E-03
**F19**	0.00E+00	0.00E+00	0.00E+00	9.44E-03
**F20**	0.00E+00	0.00E+00	0.00E+00	1.10E-03
**F21**	1.00E+02	1.00E+02	1.00E+02	0.00E+00
**F22**	0.00E+00	1.01E+02	4.91E+01	5.03E+01
**F23**	0.00E+00	3.03E+02	2.78E+02	7.17E+01
**F24**	0.00E+00	2.00E+02	1.03E+02	3.15E+01
**F25**	1.00E+02	3.98E+02	3.72E+02	7.66E+01
**F26**	0.00E+00	3.00E+02	1.94E+02	1.06E+02
**F27**	3.87E+02	3.89E+02	3.88E+02	8.09E-01
**F28**	3.00E+02	3.00E+02	3.00E+02	0.00E+00
**F29**	1.55E+02	2.32E+02	2.24E+02	1.82E+01
**F30**	3.95E+02	3.95E+02	3.95E+02	4.07E-13

**Table 7:** Results for ADMO in 30 dimensions.

Function	Best	Worst	Mean	Std
**F1**	0.00E+00	0.00E+00	0.00E+00	0.00E+00
**F3**	0.00E+00	0.00E+00	0.00E+00	0.00E+00
**F4**	5.86E+01	6.41E+01	6.00E+01	2.47E+00
**F5**	1.79E+01	4.58E+01	3.08E+01	7.53E+00
**F6**	5.00E-02	6.20E-01	1.90E-01	1.39E-01
**F7**	4.30E+01	7.29E+01	5.67E+01	7.80E+00
**F8**	2.19E+01	4.68E+01	3.37E+01	7.58E+00
**F9**	0.00E+00	5.25E+00	9.70E-01	1.21E+00
**F10**	1.28E+03	2.91E+03	1.91E+03	4.08E+02
**F11**	4.00E+00	1.29E+01	7.60E+00	2.14E+00
**F12**	1.00E-01	1.21E+03	1.06E+02	2.17E+02
**F13**	2.00E+00	2.19E+01	1.15E+01	6.47E+00
**F14**	1.00E+00	2.41E+01	5.90E+00	6.58E+00
**F15**	1.40E+00	1.16E+01	4.30E+00	2.42E+00
**F16**	9.60E+00	4.90E+02	2.02E+02	1.15E+02
**F17**	2.45E+01	1.49E+02	3.88E+01	2.14E+01
**F18**	2.30E+00	2.30E+01	2.10E+01	3.54E+00
**F19**	9.00E-01	6.50E+00	3.50E+00	1.35E+00
**F20**	6.80E+00	1.42E+02	2.53E+01	2.33E+01
**F21**	1.00E+02	2.55E+02	2.14E+02	3.86E+01
**F22**	1.00E+02	1.00E+02	1.00E+02	1.44E-13
**F23**	1.00E+02	3.87E+02	3.20E+02	1.10E+02
**F24**	4.27E+02	4.57E+02	4.40E+02	6.89E+00
**F25**	3.83E+02	3.87E+02	3.86E+02	1.24E+00
**F26**	3.00E+02	1.42E+03	3.58E+02	2.32E+02
**F27**	4.69E+02	5.08E+02	4.88E+02	9.43E+00
**F28**	3.00E+02	3.00E+02	3.00E+02	8.30E-14
**F29**	3.33E+02	4.72E+02	4.14E+02	3.84E+01
**F30**	1.94E+03	2.16E+03	1.96E+03	3.97E+01

**Table 8:** Results for ADMO in 50 dimensions.

Function	Best	Worst	Mean	Std
**F1**	6.32E+01	2.65E+07	1.30E+06	5.26E+06
**F3**	0.00E+00	1.68E+03	9.99E+01	388.15
**F4**	0.00E+00	4.51E+02	7.03E+01	82.216
**F5**	4.18E+01	1.42E+02	8.13E+01	20.86
**F6**	1.49E+00	5.92E+00	3.59E+00	1.0838
**F7**	9.27E+01	4.77E+02	1.38E+02	68.56
**F8**	3.68E+01	4.14E+02	9.16E+01	63.908
**F9**	1.24E+01	3.68E+02	1.20E+02	74.59
**F10**	3.14E+03	5.40E+03	4.28E+03	524.72
**F11**	3.52E+01	1.19E+02	7.48E+01	20.304
**F12**	1.04E+03	9.14E+03	3.50E+03	1872.3
**F13**	1.61E+02	1.05E+03	4.31E+02	185.59
**F14**	2.65E+01	4.72E+01	3.43E+01	11.276
**F15**	4.07E+01	1.03E+02	7.14E+01	25.576
**F16**	1.00E-02	9.00E-01	2.00E-02	1.87E-02
**F17**	2.41E+02	6.80E+02	5.09E+02	167.58
**F18**	2.91E+01	1.25E+03	8.67E+01	215.79
**F19**	1.84E+01	3.70E+01	2.57E+01	4.7941
**F20**	6.36E+01	4.25E+02	2.50E+02	107.28
**F21**	2.46E+02	5.85E+02	3.29E+02	143.8
**F22**	4.27E+03	5.39E+03	4.91E+03	471.25
**F23**	4.65E+02	4.91E+02	4.77E+02	9.37E+00
**F24**	5.47E+02	8.17E+02	6.08E+02	117.01
**F25**	4.58E+02	4.80E+02	4.70E+02	10.988
**F26**	3.00E+02	1.91E+03	1.53E+03	692.13
**F27**	4.96E+02	6.59E+02	5.69E+02	76.511
**F28**	0.00E+00	2.00E+02	9.00E-01	8.12E-01
**F29**	3.27E+02	5.08E+02	4.07E+02	4.48E+01
**F30**	598270	8.02E+05	6.68E+05	88961

**Table 9:** Results for ADMO in 100 dimensions.

Function	Best	Worst	Mean	Std
**F1**	6.60E+01	2.65E+07	1.30E+06	5.26E+06
**F3**	1.47E+00	1.68E+03	1.01E+02	3.89E+02
**F4**	1.17E+00	4.52E+02	7.12E+01	8.32E+01
**F5**	4.27E+01	1.43E+02	8.22E+01	2.18E+01
**F6**	2.36E+00	6.90E+00	4.47E+00	2.04E+00
**F7**	9.36E+01	4.78E+02	1.39E+02	6.95E+01
**F8**	3.77E+01	4.15E+02	9.25E+01	6.49E+01
**F9**	1.32E+01	3.69E+02	1.21E+02	7.56E+01
**F10**	3.14E+03	5.40E+03	4.28E+03	5.26E+02
**F11**	3.61E+01	1.20E+02	7.57E+01	2.13E+01
**F12**	1.04E+03	9.14E+03	3.50E+03	1.87E+03
**F13**	1.61E+02	1.05E+03	4.32E+02	1.87E+02
**F14**	2.74E+01	4.82E+01	3.52E+01	1.22E+01
**F15**	4.16E+01	1.04E+02	7.23E+01	2.65E+01
**F16**	8.80E-01	1.88E+00	8.96E-01	9.79E-01
**F17**	2.42E+02	6.81E+02	5.10E+02	1.69E+02
**F18**	3.00E+01	1.25E+03	8.76E+01	2.17E+02
**F19**	1.93E+01	3.80E+01	2.66E+01	5.75E+00
**F20**	6.45E+01	4.26E+02	2.51E+02	1.08E+02
**F21**	2.47E+02	5.86E+02	3.30E+02	1.45E+02
**F22**	4.27E+03	5.39E+03	4.91E+03	4.72E+02
**F23**	4.66E+02	4.92E+02	4.78E+02	1.03E+01
**F24**	5.47E+02	8.18E+02	6.09E+02	1.18E+02
**F25**	4.59E+02	4.81E+02	4.71E+02	1.19E+01
**F26**	3.01E+02	1.91E+03	1.53E+03	6.93E+02
**F27**	4.97E+02	6.60E+02	5.70E+02	7.75E+01
**F28**	8.70E-01	2.01E+02	1.78E+00	1.77E+00
**F29**	3.28E+02	5.09E+02	4.08E+02	4.58E+01
**F30**	5.98E+05	8.02E+05	6.68E+05	8.90E+04

Generally, the ADMO showed consistent performance for the unimodal problems (f1–f3). It successfully found the solutions for D=10, 30, and 50 but none for 100 dimensions. The mean value ranges from 0 to 3.95E+02, and the standard deviation is between 0 and 7.17E+01 for 10 dimensions. For 30 dimensions, the mean and standard deviation ranges between 0 to 1.96E+03, and 0 to 2.32E+02, respectively. The mean value for 50 dimensions ranges from 9.00E-01 to 6.68E+05, and the standard deviation is between 1.87E-02 and 5.26E+06. The performance of ADMO for simple multimodal functions (f4–f10) is competitive, as seen by the number of functions it successfully found solutions for. The ADMO found solutions for 3 of the simple multimodal functions over 51 runs and 5 functions at least once for 10 dimensions. The ADMO found solutions for 1 of the simple multimodal function for 30 and 50 dimensions, respectively. For the hybrid functions (F11–F20), the ADMO successfully found solutions for all 10 functions for 10 dimensions and none for 30, 50, and 100 dimensions.

Finally, the ADMO successfully found solutions for 4 composition functions (F21–F30) in 10 dimensions, none for 30 dimensions, and 1 for 50 dimensions. In most cases, the ADMO got trapped in solutions that are very close to the global optimal solutions, as noticed in the mean value and standard deviation ranging between 0 and 6.68E+05 across all dimensions. These values are small even for the worst returned result for all dimensions considered. It can conclusively be said that the ADMO is a stable and efficient algorithm for solving the CEC 2017 benchmark problems. Also, the results across all the dimensions considered showed that the performance of ADMO slightly decreases as the dimension increases. However, it still showed stability and robustness over the different dimensions.

#### 4.1.2. Comparative results for CEC 2017

The comparative results of the ADMO and 8 other state-of-the-art algorithms on the benchmark problems with varying dimensions of 10, 30, 50, and 100 are presented in Tables 10–13. The best and standard deviation are the only two performance metrics used, and the best-returned results are marked in boldface. In addition, the 9 metaheuristic algorithms are ranked according to the scoring metric defined in CEC 2017 technical report and presented in Table 14. The Wilcoxon signed test was also performed on the results returned by the 9 algorithms across the different dimensions considered, and the results are presented in Table 15.

**Table 10:** Comparative results for 10 dimensions.

Algorithms	ADMO		LSHADEcnEpSin		LSHADE		DMO		LSHADE_SPACMA		UMOEA		WOA		AOA		CPSOGSA	
Function	Best	Std	Best	Std	Best	Std	Best	Std	Best	Std	Best	Std	Best	Std	Best	Std	Best	Std
**F1**	0.00E+00	0.00E+00	0.00E+00	0.00E+00	0.00E+00	0.00E+00	9.70E-01	3.14E+03	0.00E+00	0.00E+00	0.00E+00	0.00E+00	4.07E+07	3.10E+03	2.21E+03	2.07E+03	1.00E-01	3.78E+03
**F3**	0.00E+00	0.00E+00	0.00E+00	0.00E+00	0.00E+00	0.00E+00	0.00E+00	2.28E-02	0.00E+00	0.00E+00	0.00E+00	0.00E+00	4.28E+03	1.08E-01	1.00E-02	6.05E-03	0.00E+00	0.00E+00
**F4**	0.00E+00	0.00E+00	0.00E+00	0.00E+00	0.00E+00	0.00E+00	1.00E-01	1.21E+00	0.00E+00	0.00E+00	0.00E+00	0.00E+00	1.54E+01	2.23E+01	9.00E-02	1.24E+01	0.00E+00	1.00E+01
**F5**	0.00E+00	6.22E-01	0.00E+00	8.71E-01	9.90E-01	8.29E-01	8.02E+00	1.11E+01	0.00E+00	7.15E-01	0.00E+00	3.85E-13	1.80E+01	1.39E+01	2.49E+01	1.38E+01	1.59E+01	1.20E+01
**F6**	0.00E+00	5.77E-10	0.00E+00	0.00E+00	0.00E+00	0.00E+00	1.50E-01	5.42E+00	0.00E+00	0.00E+00	0.00E+00	0.00E+00	1.54E+01	1.15E+01	1.79E+01	7.87E+00	0.00E+00	1.11E+01
**F7**	1.00E+00	3.40E+00	1.07E+01	4.89E-01	1.07E+01	8.05E-01	2.49E+01	1.23E+01	1.05E+01	3.65E-01	1.04E+01	3.32E-01	3.14E+01	1.97E+01	4.57E+01	1.53E+01	1.77E+01	1.28E+01
**F8**	0.00E+00	4.80E-01	0.00E+00	9.24E-01	9.90E-01	9.46E-01	7.04E+00	5.75E+00	0.00E+00	9.25E-01	0.00E+00	5.59E-01	1.48E+01	1.07E+01	1.59E+01	6.26E+00	9.95E+00	1.14E+01
**F9**	0.00E+00	0.00E+00	0.00E+00	0.00E+00	0.00E+00	0.00E+00	4.00E-02	1.77E+02	0.00E+00	0.00E+00	0.00E+00	0.00E+00	1.06E+02	3.01E+02	1.67E+02	1.74E+02	0.00E+00	5.02E+02
**F10**	3.00E-01	3.44E+01	1.00E-01	3.88E+01	3.00E-01	4.08E+01	2.42E+02	2.82E+02	2.00E-01	5.81E+01	4.00E-01	3.55E+01	6.10E+02	2.59E+02	2.56E+02	2.93E+02	1.52E+01	3.45E+02
**F11**	0.00E+00	0.00E+00	0.00E+00	0.00E+00	0.00E+00	1.22E-02	1.02E+01	1.15E+01	0.00E+00	1.79E-01	0.00E+00	1.79E-01	2.65E+01	6.35E+01	1.27E+01	5.50E+00	1.50E+01	4.94E+01
**F12**	0.00E+00	1.03E-05	2.00E-01	1.30E+02	0.00E+00	7.71E+01	1.98E+03	1.48E+05	0.00E+00	9.38E+01	0.00E+00	5.70E+01	2.03E+04	2.53E+06	1.25E+04	2.45E+04	1.08E+03	1.73E+04
**F13**	0.00E+00	3.34E-02	0.00E+00	2.71E+00	0.00E+00	2.61E+00	3.80E+02	8.45E+03	0.00E+00	2.87E+00	0.00E+00	2.05E+00	1.57E+03	1.20E+04	6.55E+02	9.65E+03	2.65E+02	4.62E+03
**F14**	0.00E+00	0.00E+00	0.00E+00	1.79E-01	0.00E+00	4.67E-01	3.25E+01	2.03E+01	0.00E+00	7.92E+00	0.00E+00	6.03E-01	5.23E+01	2.70E+01	1.22E+02	9.26E+03	2.90E+01	3.77E+01
**F15**	0.00E+00	9.99E-04	0.00E+00	2.98E-01	0.00E+00	1.45E-01	1.06E+01	4.64E+01	0.00E+00	2.71E-01	0.00E+00	1.47E-01	5.07E+02	7.60E+01	9.23E+01	4.13E+03	1.78E+01	5.49E+01
**F16**	0.00E+00	9.50E-02	0.00E+00	3.53E-01	0.00E+00	1.75E-01	4.10E+00	1.34E+02	1.00E-01	5.09E-01	0.00E+00	1.55E-01	3.73E+01	9.05E+01	5.30E+00	1.28E+02	2.20E+00	1.39E+02
**F17**	0.00E+00	4.50E-01	0.00E+00	4.97E+00	0.00E+00	1.25E-01	2.56E+01	2.78E+01	0.00E+00	4.97E+00	0.00E+00	1.68E-01	3.19E+01	2.39E+01	3.77E+01	8.31E+01	2.03E+01	5.58E+01
**F18**	0.00E+00	1.06E-03	0.00E+00	9.73E+00	0.00E+00	2.01E-01	1.14E+02	8.56E+03	0.00E+00	9.99E+00	0.00E+00	1.89E-01	2.01E+03	1.25E+04	1.61E+03	9.45E+03	5.66E+02	5.31E+03
**F19**	0.00E+00	9.44E-03	0.00E+00	3.38E-02	0.00E+00	6.02E-03	1.35E+01	8.18E+02	0.00E+00	4.76E-01	0.00E+00	7.88E-03	4.20E+02	7.38E+03	2.30E+01	1.22E+04	6.60E+00	3.70E+01
**F20**	0.00E+00	1.10E-03	0.00E+00	1.55E-01	0.00E+00	1.06E-01	2.63E+01	3.59E+01	0.00E+00	8.50E+00	0.00E+00	0.00E+00	3.49E+01	3.29E+01	4.66E+01	6.17E+01	3.59E+01	8.64E+01
**F21**	1.00E+02	0.00E+00	1.00E+02	5.11E+01	1.00E+02	5.06E+01	1.00E+02	6.97E+01	1.00E+02	1.90E+01	1.00E+02	5.05E+01	1.14E+02	7.00E+01	1.00E+02	4.54E+01	1.00E+02	6.18E+01
**F22**	0.00E+00	5.03E+01	1.00E+02	8.73E-02	1.00E+02	6.19E-02	5.70E+01	1.52E+01	1.00E+02	1.65E-01	0.00E+00	1.80E+01	1.37E+02	1.55E+01	4.94E+01	9.84E+01	1.01E+02	1.41E+00
**F23**	0.00E+00	7.17E+01	3.00E+02	1.52E+00	3.00E+02	1.32E+00	3.08E+02	2.34E+01	3.00E+02	1.33E+00	3.00E+02	8.20E-01	3.36E+02	1.56E+01	3.48E+02	2.54E+01	3.13E+02	8.82E+00
**F24**	0.00E+00	3.15E+01	1.00E+02	7.82E+01	1.00E+02	7.88E+01	1.00E+02	5.69E+01	0.00E+00	1.10E+02	2.33E+01	1.10E+02	1.65E+02	9.16E+01	1.00E+02	1.18E+02	3.41E+02	1.17E+01
**F25**	1.00E+02	7.66E+01	3.98E+02	2.31E+01	3.98E+02	2.23E+01	4.00E+02	2.09E+01	3.98E+02	2.31E+01	3.98E+02	2.25E+01	2.83E+02	2.58E+01	3.98E+02	3.36E+01	3.98E+02	3.30E+01
**F26**	0.00E+00	1.06E+02	3.00E+02	0.00E+00	3.00E+02	0.00E+00	1.10E+00	2.44E+02	3.00E+02	0.00E+00	0.00E+00	6.68E+01	2.53E+02	3.81E+02	8.00E-01	3.69E+02	2.00E+02	3.39E+02
**F27**	3.87E+02	8.09E-01	3.87E+02	2.15E+00	3.89E+02	2.28E-01	3.96E+02	3.18E+01	3.89E+02	1.83E+00	3.89E+02	1.75E-01	3.88E+02	2.46E+01	4.09E+02	3.46E+01	3.90E+02	1.99E+01
**F28**	3.00E+02	0.00E+00	3.00E+02	1.40E+02	3.00E+02	1.50E+02	3.00E+02	1.26E+02	3.00E+02	1.04E+02	3.00E+02	6.58E+01	4.60E+02	9.73E+01	3.00E+02	1.26E+02	3.00E+02	1.38E+02
**F29**	1.55E+02	1.82E+01	2.26E+02	2.70E+00	2.30E+02	2.46E+00	2.49E+02	5.94E+01	2.26E+02	3.98E+00	2.25E+02	3.81E+00	2.74E+02	7.06E+01	2.84E+02	9.67E+01	2.40E+02	6.13E+01
**F30**	3.95E+02	4.07E-13	3.91E+02	3.39E+05	3.95E+02	2.46E+05	1.14E+03	2.12E+05	3.95E+02	2.64E+05	3.95E+02	2.23E+01	1.89E+04	1.48E+05	7.20E+03	5.79E+04	9.92E+02	8.06E+05

**Table 11:** Comparative results for 30 dimensions.

Algorithms	ADMO		LSHADEcnEpSin		LSHADE		DMO		LSHADE_SPACMA		UMOEA		WOA		AOA		CPSOGSA	
Function	Best	Std	Best	Std	Best	Std	Best	Std	Best	Std	Best	Std	Best	Std	Best	Std	Best	Std
**F1**	0.00E+00	0.00E+00	0.00E+00	8.61E-15	0.00E+00	1.13E-14	1.20E+06	1.01E+06	0.00E+00	0.00E+00	0.00E+00	2.59E-15	2.13E+10	4.85E+03	1.26E+10	2.96E+09	1.59E+00	7.34E+03
**F3**	0.00E+00	0.00E+00	0.00E+00	1.77E-13	0.00E+00	2.94E-14	1.07E+01	5.18E+00	0.00E+00	1.77E+04	0.00E+00	4.06E-12	5.61E+04	2.00E+04	3.31E+04	6.29E+03	0.00E+00	4.28E-14
**F4**	5.86E+01	2.47E+00	0.00E+00	2.39E+01	0.00E+00	1.50E+01	6.54E+01	1.88E+01	0.00E+00	2.73E+01	0.00E+00	1.20E+00	2.43E+03	2.42E+01	8.48E+02	9.07E+02	1.00E+00	2.86E+01
**F5**	1.79E+01	7.53E+00	8.95E+00	3.30E+00	7.96E+00	2.88E+00	1.16E+02	3.19E+01	7.96E+00	3.16E+00	0.00E+00	1.87E+00	2.45E+02	5.34E+01	1.36E+02	3.84E+01	1.18E+02	5.83E+01
**F6**	5.00E-02	1.39E-01	0.00E+00	3.70E-04	0.00E+00	8.93E-03	3.73E+01	6.31E+00	0.00E+00	1.15E-03	0.00E+00	2.96E-03	5.10E+01	9.04E+00	4.14E+01	6.29E+00	1.96E+01	1.24E+01
**F7**	4.30E+01	7.80E+00	4.06E+01	2.62E+00	3.89E+01	2.34E+00	3.03E+02	6.70E+01	3.87E+01	3.14E+00	3.30E+01	1.94E+00	5.61E+02	7.03E+01	4.27E+02	5.89E+01	1.27E+02	5.73E+01
**F8**	2.19E+01	7.58E+00	9.95E+00	3.26E+00	8.95E+00	3.80E+00	1.03E+02	1.83E+01	6.96E+00	4.19E+00	9.90E-01	1.71E+00	2.12E+02	3.17E+01	1.02E+02	2.58E+01	1.23E+02	4.15E+01
**F9**	0.00E+00	1.21E+00	0.00E+00	9.81E-01	0.00E+00	6.82E-01	3.12E+03	4.77E+02	0.00E+00	5.15E-01	0.00E+00	1.19E+00	5.55E+03	1.53E+03	2.73E+03	6.14E+02	2.61E+03	2.15E+03
**F10**	1.28E+03	4.08E+02	1.17E+03	1.94E+02	1.08E+03	2.50E+02	2.34E+03	7.02E+02	1.11E+03	2.69E+02	1.21E+03	1.85E+02	5.39E+03	6.48E+02	2.63E+03	5.60E+02	2.68E+03	6.78E+02
**F11**	4.00E+00	2.14E+00	1.06E+01	2.42E+01	6.10E+00	2.57E+01	5.62E+01	4.75E+01	6.20E+00	3.00E+01	8.00E+00	1.83E+01	1.17E+03	6.65E+01	1.68E+02	6.20E+01	5.84E+01	7.89E+01
**F12**	1.00E-01	2.17E+02	7.48E+02	3.76E+02	2.36E+02	4.23E+02	1.54E+06	2.20E+06	8.17E+02	2.86E+02	3.57E+02	3.78E+02	1.47E+09	7.57E+06	1.52E+06	2.03E+08	9.97E+03	3.69E+04
**F13**	2.00E+00	6.47E+00	1.09E+02	3.98E+02	1.19E+01	1.52E+01	1.65E+04	3.49E+04	5.37E+01	1.67E+02	1.69E+01	2.13E+01	9.33E+06	1.08E+05	1.83E+04	1.46E+04	2.35E+03	2.33E+04
**F14**	1.00E+00	6.58E+00	2.40E+01	2.35E+01	2.31E+01	6.13E+00	6.30E+02	8.31E+03	3.00E+01	2.49E+01	1.21E+01	6.81E+00	1.61E+05	1.33E+05	2.54E+03	1.10E+04	1.89E+02	2.97E+02
**F15**	1.40E+00	2.42E+00	2.92E+01	5.65E+01	4.30E+00	1.76E+01	2.83E+03	9.99E+03	1.51E+01	4.58E+01	8.40E+00	1.38E+01	2.59E+04	5.16E+04	1.13E+04	1.00E+04	2.68E+02	1.47E+04
**F16**	9.60E+00	1.15E+02	4.90E+00	1.82E+02	1.39E+01	1.25E+02	6.63E+02	2.93E+02	4.50E+00	1.50E+02	1.37E+01	1.21E+02	1.30E+03	3.79E+02	7.23E+02	4.00E+02	5.08E+02	3.59E+02
**F17**	2.45E+01	2.14E+01	1.47E+01	1.10E+01	3.04E+01	1.20E+01	8.33E+01	2.14E+02	1.55E+01	2.72E+01	2.70E+01	2.15E+01	2.22E+02	2.28E+02	4.82E+02	2.52E+02	3.86E+02	2.08E+02
**F18**	2.30E+00	3.54E+00	4.39E+01	8.12E+01	2.36E+01	4.12E+01	3.17E+04	1.83E+05	6.49E+01	7.98E+01	2.31E+01	3.63E+01	2.66E+05	7.43E+05	4.28E+04	6.51E+04	5.94E+03	1.99E+04
**F19**	9.00E-01	1.35E+00	1.73E+01	3.07E+01	5.30E+00	1.13E+01	2.18E+03	1.58E+04	3.04E+01	5.05E+01	4.00E+00	9.58E+00	1.25E+04	3.60E+05	9.23E+04	1.16E+05	6.66E+02	1.76E+04
**F20**	6.80E+00	2.33E+01	1.08E+01	3.76E+01	8.50E+00	4.58E+01	1.06E+02	1.69E+02	2.52E+01	5.87E+01	2.99E+01	5.53E+01	3.65E+02	2.09E+02	3.15E+02	1.61E+02	3.18E+02	2.12E+02
**F21**	1.00E+02	3.86E+01	2.11E+02	3.44E+00	2.10E+02	2.32E+00	1.01E+02	6.24E+01	2.11E+02	3.25E+00	1.00E+02	3.21E+01	4.35E+02	5.92E+01	3.29E+02	4.98E+01	3.24E+02	4.75E+01
**F22**	1.00E+02	1.44E-13	1.00E+02	6.18E-01	1.00E+02	4.42E-01	1.14E+02	2.25E+03	1.00E+02	1.17E-13	1.00E+02	0.00E+00	3.19E+03	2.01E+03	2.89E+03	6.83E+02	1.00E+02	1.65E+03
**F23**	1.00E+02	1.10E+02	3.47E+02	6.50E+00	3.49E+02	5.34E+00	5.38E+02	9.24E+01	3.48E+02	6.80E+00	3.42E+02	6.57E+00	7.47E+02	1.00E+02	7.46E+02	1.02E+02	4.67E+02	7.55E+01
**F24**	4.27E+02	6.89E+00	4.31E+02	4.69E+00	4.27E+02	4.09E+00	6.82E+02	1.17E+02	4.26E+02	4.78E+00	2.00E+02	4.11E+01	8.36E+02	8.86E+01	9.66E+02	1.32E+02	5.84E+02	7.58E+01
**F25**	3.83E+02	1.24E+00	3.87E+02	3.53E-01	3.87E+02	3.37E-01	3.84E+02	1.60E+01	3.87E+02	3.57E-01	3.87E+02	2.64E-01	1.13E+03	2.38E+01	6.05E+02	1.29E+02	3.84E+02	9.97E+00
**F26**	3.00E+02	2.32E+02	9.86E+02	8.49E+01	9.31E+02	5.87E+01	2.33E+02	1.03E+03	9.52E+02	7.73E+01	2.00E+02	3.54E+02	4.90E+03	1.53E+03	4.54E+03	6.16E+02	2.00E+02	1.21E+03
**F27**	4.69E+02	9.43E+00	4.91E+02	7.01E+00	4.92E+02	7.88E+00	5.03E+02	3.84E+01	4.94E+02	7.24E+00	4.80E+02	1.27E+01	5.05E+02	6.73E+01	8.93E+02	2.43E+02	5.24E+02	4.67E+01
**F28**	3.00E+02	8.30E-14	3.00E+02	6.18E+01	3.00E+02	6.47E+01	3.96E+02	2.43E+01	3.00E+02	5.78E+01	3.00E+02	4.96E+01	5.00E+02	3.36E+01	8.79E+02	5.18E+02	3.00E+02	5.48E+01
**F29**	3.33E+02	3.84E+01	4.15E+02	2.89E+01	3.66E+02	3.20E+01	6.05E+02	3.01E+02	4.21E+02	6.24E+01	4.13E+02	2.29E+01	1.57E+03	3.68E+02	1.57E+03	5.42E+02	5.89E+02	3.03E+02
**F30**	1.94E+03	3.97E+01	1.62E+03	2.65E+02	1.94E+03	1.01E+02	8.73E+04	1.64E+05	1.98E+03	1.86E+02	1.97E+03	9.48E+01	2.72E+06	1.65E+06	1.22E+06	3.76E+07	5.93E+03	7.49E+03

**Table 12:** Comparative results for 50 dimensions.

Algorithms	ADMO		LSHADEcnEpSin		LSHADE		DMO		LSHADE_SPACMA		UMOEA		WOA		AOA		CPSOGSA	
Function	Best	Std	Best	Std	Best	Std	Best	Std	Best	Std	Best	Std	Best	Std	Best	Std	Best	Std
**F1**	6.32E+01	5.26E+06	0.00E+00	8.24E-10	0.00E+00	2.06E-14	8.84E+06	3.40E+06	0.00E+00	0.00E+00	0.00E+00	9.71E-15	6.72E+10	1.18E+04	7.41E+10	4.89E+09	2.70E+01	1.11E+04
**F3**	0.00E+00	3.88E+02	0.00E+00	3.30E-13	0.00E+00	3.44E+04	4.07E+01	1.60E+01	0.00E+00	4.22E+04	0.00E+00	3.92E-11	1.51E+05	2.24E+03	1.17E+05	1.67E+04	0.00E+00	1.33E-13
**F4**	0.00E+00	8.22E+01	0.00E+00	4.61E+01	0.00E+00	5.09E+01	3.26E+01	5.00E+01	0.00E+00	4.58E+01	0.00E+00	1.69E+00	1.95E+04	6.39E+01	1.01E+04	2.34E+03	2.00E-02	4.62E+01
**F5**	4.18E+01	2.09E+01	2.39E+01	7.36E+00	2.29E+01	5.97E+00	2.90E+02	3.09E+01	1.99E+01	7.96E+00	1.19E+01	5.47E+00	5.51E+02	5.90E+01	4.04E+02	3.54E+01	3.12E+02	6.34E+01
**F6**	1.49E+00	1.08E+00	2.00E-02	1.91E-01	1.00E-02	1.14E-01	5.41E+01	4.46E+00	0.00E+00	6.46E-02	0.00E+00	7.86E-02	7.45E+01	8.33E+00	6.63E+01	3.95E+00	3.97E+01	8.27E+00
**F7**	9.27E+01	6.86E+01	7.10E+01	7.04E+00	8.14E+01	7.72E+00	7.83E+02	7.91E+01	7.29E+01	8.56E+00	6.73E+01	1.06E+01	9.47E+02	1.16E+02	9.88E+02	4.62E+01	2.72E+02	9.63E+01
**F8**	3.68E+01	6.39E+01	2.99E+01	7.94E+00	1.99E+01	4.45E+00	2.49E+02	3.65E+01	2.69E+01	5.79E+00	8.95E+00	5.71E+00	6.13E+02	5.07E+01	4.41E+02	4.59E+01	3.54E+02	6.95E+01
**F9**	1.24E+01	7.46E+01	3.63E+00	2.39E+01	4.90E+00	1.41E+01	1.05E+04	8.12E+02	1.54E+00	1.20E+01	5.54E+00	1.69E+01	2.24E+04	4.38E+03	1.25E+04	2.43E+03	1.08E+04	4.46E+03
**F10**	3.14E+03	5.25E+02	2.52E+03	2.88E+02	2.40E+03	3.22E+02	4.23E+03	9.58E+02	2.58E+03	2.99E+02	2.48E+03	2.53E+02	1.06E+04	1.10E+03	8.10E+03	6.79E+02	5.38E+03	8.39E+02
**F11**	3.52E+01	2.03E+01	8.56E+01	4.68E+01	8.69E+01	4.95E+01	1.54E+02	5.53E+01	8.89E+01	4.59E+01	4.71E+01	4.49E+01	1.34E+04	7.04E+01	1.30E+03	5.34E+02	1.79E+02	6.05E+01
**F12**	1.04E+03	1.87E+03	1.79E+03	4.65E+02	8.92E+02	4.85E+02	9.79E+06	1.71E+07	9.56E+02	5.01E+02	1.04E+03	4.44E+02	1.21E+10	2.26E+07	6.39E+09	7.23E+09	1.41E+05	6.53E+05
**F13**	1.61E+02	1.86E+02	1.26E+03	8.59E+02	7.56E+01	1.48E+02	4.03E+04	1.22E+05	5.10E+02	9.48E+02	1.06E+02	2.44E+02	1.72E+09	1.22E+05	5.24E+04	9.42E+03	5.46E+03	1.38E+04
**F14**	2.65E+01	1.13E+01	1.65E+02	1.37E+02	1.48E+02	4.96E+01	1.81E+04	1.26E+05	2.64E+02	4.18E+01	1.25E+02	3.83E+01	4.13E+06	4.76E+04	7.68E+03	1.63E+04	5.65E+02	4.20E+02
**F15**	4.07E+01	2.56E+01	2.33E+02	1.45E+02	1.11E+02	9.66E+01	1.75E+04	1.50E+04	3.30E+02	7.52E+01	9.82E+01	1.01E+02	4.62E+08	2.75E+04	1.77E+04	7.29E+03	1.41E+03	1.18E+04
**F16**	1.00E-02	1.87E-02	1.00E-02	1.54E-03	1.00E-02	1.78E-01	1.54E+03	3.25E+02	1.27E+02	1.68E+02	1.60E+02	2.37E+01	1.33E+02	8.78E+01	1.87E+03	2.20E+02	2.15E+01	1.23E+02
**F17**	2.41E+02	1.68E+02	8.81E+01	2.18E+02	2.90E+02	1.45E+02	1.77E+03	2.88E+02	2.64E+02	1.14E+02	2.63E+02	1.41E+02	1.68E+03	1.65E+02	1.65E+03	2.47E+02	1.53E+03	3.81E+02
**F18**	2.91E+01	2.16E+02	8.75E+01	1.49E+02	7.11E+01	1.10E+02	1.52E+05	3.04E+05	7.31E+01	1.43E+02	2.93E+01	5.72E+01	6.03E+06	9.20E+05	1.41E+05	6.63E+05	7.46E+03	1.29E+04
**F19**	1.84E+01	4.79E+00	9.06E+01	6.63E+01	5.02E+01	5.40E+01	3.98E+03	3.66E+04	7.72E+01	5.77E+01	6.86E+01	4.00E+01	3.73E+07	3.97E+05	4.01E+05	1.40E+04	5.54E+02	1.42E+04
**F20**	6.36E+01	1.07E+02	8.76E+01	1.18E+02	5.70E+01	1.25E+02	7.67E+02	2.50E+02	9.13E+01	1.23E+02	5.39E+01	1.07E+02	1.33E+03	3.17E+02	7.49E+02	2.80E+02	9.42E+02	3.15E+02
**F21**	2.46E+02	1.44E+02	2.34E+02	5.77E+00	2.34E+02	2.78E+00	5.79E+02	1.19E+02	2.31E+02	7.25E+00	2.20E+02	4.62E+00	8.94E+02	5.18E+01	8.02E+02	3.26E+01	5.09E+02	1.05E+02
**F22**	4.27E+03	4.71E+02	1.00E+02	1.61E+03	1.04E+02	2.04E+03	6.62E+03	1.00E+03	1.00E+02	2.48E+03	3.32E+03	3.86E+02	1.29E+04	1.50E+03	1.09E+04	8.25E+02	6.36E+03	8.36E+02
**F23**	4.65E+02	9.37E+00	4.55E+02	5.88E+00	4.44E+02	7.33E+00	9.16E+02	1.07E+02	4.53E+02	8.47E+00	4.39E+02	3.17E+00	1.36E+03	1.47E+02	1.36E+03	2.70E+02	1.10E+03	1.85E+02
**F24**	5.47E+02	1.17E+02	5.35E+02	9.75E+00	5.24E+02	4.20E+00	1.36E+03	1.08E+02	5.30E+02	1.08E+01	5.17E+02	5.13E+00	1.66E+03	8.03E+01	1.89E+03	2.20E+02	1.10E+03	5.32E+01
**F25**	4.58E+02	1.10E+01	4.61E+02	5.10E+01	4.80E+02	4.93E+01	5.33E+02	2.87E+01	5.30E+02	1.70E+01	4.80E+02	2.50E+01	1.07E+04	1.86E+01	5.28E+03	8.78E+02	5.40E+02	1.40E+01
**F26**	3.00E+02	6.92E+02	1.45E+03	2.06E+02	1.32E+03	2.36E+02	3.19E+02	3.61E+03	1.32E+03	1.16E+02	3.00E+02	1.07E-06	1.19E+04	1.72E+03	1.13E+04	6.05E+02	7.31E+03	1.44E+03
**F27**	4.96E+02	7.65E+01	5.44E+02	6.77E+01	5.49E+02	6.92E+01	9.24E+02	1.40E+02	5.38E+02	5.24E+01	5.81E+02	2.13E+01	1.79E+03	4.27E+02	2.95E+03	5.13E+02	9.11E+02	2.56E+02
**F28**	0.00E+00	8.12E-01	2.82E+01	3.31E+01	3.30E+01	4.55E+01	3.23E+03	2.14E+02	4.53E+02	4.38E+01	4.39E+01	2.57E+01	2.80E+03	3.25E+02	3.89E+02	3.40E+01	2.54E+02	4.31E+02
**F29**	3.27E+02	4.48E+01	3.45E+02	3.51E+01	3.65E+02	3.56E+01	6.44E+02	2.70E+02	3.69E+02	3.58E+01	3.88E+02	2.71E+01	1.40E+03	3.25E+02	1.57E+03	4.51E+02	6.13E+02	2.82E+02
**F30**	5.98E+05	8.90E+04	5.79E+05	2.44E+05	7.05E+05	1.40E+05	3.39E+06	9.77E+05	6.28E+05	1.02E+05	5.90E+05	5.75E+04	6.14E+08	5.52E+06	1.69E+08	4.49E+07	7.68E+05	6.95E+05

**Table 13:** Comparative results for 100 dimensions.

Algorithms	ADMO		LSHADEcnEpSin		LSHADE		DMO		LSHADE_SPACMA		UMOEA		WOA		AOA		CPSOGSA	
Function	Best	Std	Best	Std	Best	Std	Best	Std	Best	Std	Best	Std	Best	Std	Best	Std	Best	Std
**F1**	5.26E+06	6.90E+01	6.61E+01	5.26E+06	6.64E+01	5.06E+06	6.69E+01	5.26E+06	6.62E+01	5.26E+06	6.75E+01	5.26E+06	6.81E+01	5.26E+06	6.75E+01	5.26E+06	6.81E+01	5.26E+06
**F3**	3.89E+02	4.46E+00	1.59E+00	3.89E+02	1.83E+00	3.90E+02	1.83E+00	3.90E+02	1.60E+00	3.90E+02	2.90E+00	3.90E+02	3.50E+00	3.91E+02	2.90E+00	3.90E+02	3.50E+00	3.91E+02
**F4**	8.31E+01	4.16E+00	1.29E+00	8.36E+01	1.53E+00	8.40E+01	1.53E+00	8.40E+01	1.30E+00	8.41E+01	2.60E+00	8.43E+01	3.20E+00	8.52E+01	2.60E+00	8.43E+01	3.20E+00	8.52E+01
**F5**	2.17E+01	4.57E+01	4.28E+01	2.22E+01	4.30E+01	2.26E+01	4.30E+01	2.26E+01	4.28E+01	2.28E+01	4.41E+01	2.30E+01	4.47E+01	2.38E+01	4.41E+01	2.30E+01	4.47E+01	2.38E+01
**F6**	1.95E+00	5.35E+00	2.48E+00	2.42E+00	2.72E+00	2.84E+00	2.72E+00	2.84E+00	2.49E+00	2.98E+00	3.79E+00	3.17E+00	4.39E+00	4.06E+00	3.79E+00	3.17E+00	4.39E+00	4.06E+00
**F7**	6.94E+01	9.66E+01	9.37E+01	6.99E+01	9.39E+01	7.03E+01	9.39E+01	7.03E+01	9.37E+01	7.05E+01	9.50E+01	7.07E+01	9.56E+01	7.15E+01	9.50E+01	7.07E+01	9.56E+01	7.15E+01
**F8**	6.48E+01	4.07E+01	3.78E+01	6.52E+01	3.80E+01	6.57E+01	3.80E+01	6.57E+01	3.78E+01	6.58E+01	3.91E+01	6.60E+01	3.97E+01	6.69E+01	3.91E+01	6.60E+01	3.97E+01	6.69E+01
**F9**	7.55E+01	1.62E+01	1.34E+01	7.59E+01	1.36E+01	7.64E+01	1.36E+01	7.64E+01	1.34E+01	7.65E+01	1.47E+01	7.67E+01	1.53E+01	7.76E+01	1.47E+01	7.67E+01	1.53E+01	7.76E+01
**F10**	5.26E+02	3.14E+03	3.14E+03	5.26E+02	3.14E+03	5.26E+02	3.14E+03	5.26E+02	3.14E+03	5.27E+02	3.14E+03	5.27E+02	3.14E+03	5.28E+02	3.14E+03	5.27E+02	3.14E+03	5.28E+02
**F11**	2.12E+01	3.91E+01	3.62E+01	2.16E+01	3.64E+01	2.21E+01	3.64E+01	2.21E+01	3.62E+01	2.22E+01	3.75E+01	2.24E+01	3.81E+01	2.33E+01	3.75E+01	2.24E+01	3.81E+01	2.33E+01
**F12**	1.87E+03	1.04E+03	1.04E+03	1.87E+03	1.04E+03	1.87E+03	1.04E+03	1.87E+03	1.04E+03	1.87E+03	1.04E+03	1.87E+03	1.04E+03	1.88E+03	1.04E+03	1.87E+03	1.04E+03	1.88E+03
**F13**	1.86E+02	1.64E+02	1.62E+02	1.87E+02	1.62E+02	1.87E+02	1.62E+02	1.87E+02	1.62E+02	1.87E+02	1.63E+02	1.88E+02	1.64E+02	1.89E+02	1.63E+02	1.88E+02	1.64E+02	1.89E+02
**F14**	1.21E+01	3.04E+01	2.75E+01	1.26E+01	2.77E+01	1.30E+01	2.77E+01	1.30E+01	2.75E+01	1.32E+01	2.88E+01	1.34E+01	2.94E+01	1.43E+01	2.88E+01	1.34E+01	2.94E+01	1.43E+01
**F15**	2.64E+01	4.46E+01	4.17E+01	2.69E+01	4.19E+01	2.73E+01	4.19E+01	2.73E+01	4.17E+01	2.75E+01	4.30E+01	2.77E+01	4.36E+01	2.86E+01	4.30E+01	2.77E+01	4.36E+01	2.86E+01
**F16**	8.89E-01	3.87E+00	1.00E+00	1.36E+00	1.24E+00	1.78E+00	1.24E+00	1.78E+00	1.01E+00	1.92E+00	2.31E+00	2.11E+00	2.91E+00	3.00E+00	2.31E+00	2.11E+00	2.91E+00	3.00E+00
**F17**	1.68E+02	2.45E+02	2.42E+02	1.69E+02	2.42E+02	1.69E+02	2.42E+02	1.69E+02	2.42E+02	1.69E+02	2.43E+02	1.70E+02	2.44E+02	1.71E+02	2.43E+02	1.70E+02	2.44E+02	1.71E+02
**F18**	2.17E+02	3.30E+01	3.01E+01	2.17E+02	3.03E+01	2.18E+02	3.03E+01	2.18E+02	3.01E+01	2.18E+02	3.14E+01	2.18E+02	3.20E+01	2.19E+02	3.14E+01	2.18E+02	3.20E+01	2.19E+02
**F19**	5.66E+00	2.23E+01	1.94E+01	6.13E+00	1.96E+01	6.55E+00	1.96E+01	6.55E+00	1.94E+01	6.69E+00	2.07E+01	6.88E+00	2.13E+01	7.77E+00	2.07E+01	6.88E+00	2.13E+01	7.77E+00
**F20**	1.08E+02	6.75E+01	6.46E+01	1.09E+02	6.48E+01	1.09E+02	6.48E+01	1.09E+02	6.46E+01	1.09E+02	6.59E+01	1.09E+02	6.65E+01	1.10E+02	6.59E+01	1.09E+02	6.65E+01	1.10E+02
**F21**	1.45E+02	2.50E+02	2.47E+02	1.45E+02	2.47E+02	1.46E+02	2.47E+02	1.46E+02	2.47E+02	1.46E+02	2.48E+02	1.46E+02	2.49E+02	1.47E+02	2.48E+02	1.46E+02	2.49E+02	1.47E+02
**F22**	4.72E+02	4.27E+03	4.27E+03	4.73E+02	4.27E+03	4.73E+02	4.27E+03	4.73E+02	4.27E+03	4.73E+02	4.27E+03	4.73E+02	4.27E+03	4.74E+02	4.27E+03	4.73E+02	4.27E+03	4.74E+02
**F23**	1.02E+01	4.69E+02	4.66E+02	1.07E+01	4.66E+02	1.11E+01	4.66E+02	1.11E+01	4.66E+02	1.13E+01	4.67E+02	1.15E+01	4.68E+02	1.24E+01	4.67E+02	1.15E+01	4.68E+02	1.24E+01
**F24**	1.18E+02	5.50E+02	5.48E+02	1.18E+02	5.48E+02	1.19E+02	5.48E+02	1.19E+02	5.48E+02	1.19E+02	5.49E+02	1.19E+02	5.50E+02	1.20E+02	5.49E+02	1.19E+02	5.50E+02	1.20E+02
**F25**	1.19E+01	4.62E+02	4.59E+02	1.23E+01	4.60E+02	1.27E+01	4.60E+02	1.27E+01	4.59E+02	1.29E+01	4.61E+02	1.31E+01	4.61E+02	1.40E+01	4.61E+02	1.31E+01	4.61E+02	1.40E+01
**F26**	6.93E+02	3.04E+02	3.01E+02	6.93E+02	3.01E+02	6.94E+02	3.01E+02	6.94E+02	3.01E+02	6.94E+02	3.02E+02	6.94E+02	3.03E+02	6.95E+02	3.02E+02	6.94E+02	3.03E+02	6.95E+02
**F27**	7.74E+01	5.00E+02	4.97E+02	7.79E+01	4.98E+02	7.83E+01	4.98E+02	7.83E+01	4.97E+02	7.84E+01	4.99E+02	7.86E+01	4.99E+02	7.95E+01	4.99E+02	7.86E+01	4.99E+02	7.95E+01
**F28**	1.68E+00	3.86E+00	9.90E-01	2.15E+00	1.23E+00	2.57E+00	1.23E+00	2.57E+00	1.00E+00	2.71E+00	2.30E+00	2.90E+00	2.90E+00	3.79E+00	2.30E+00	2.90E+00	2.90E+00	3.79E+00
**F29**	4.57E+01	3.31E+02	3.28E+02	4.61E+01	3.29E+02	4.66E+01	3.29E+02	4.66E+01	3.28E+02	4.67E+01	3.30E+02	4.69E+01	3.30E+02	4.78E+01	3.30E+02	4.69E+01	3.30E+02	4.78E+01
**F30**	8.90E+04	5.98E+05	5.98E+05	8.90E+04	5.98E+05	8.90E+04	5.98E+05	8.90E+04	5.98E+05	8.90E+04	5.98E+05	8.90E+04	5.98E+05	8.90E+04	5.98E+05	8.90E+04	5.98E+05	8.90E+04

**Table 14:** Ranking based on scoring format defined in CEC 2017 technical report.

Algorithm	Score1	Score2	Score	Rank
**ADMO**	49.98	47.03	97.01	1
**DMO**	2.50	29.40	31.9	7
**LSHADEcnEpSin**	49.90	47.01	96.91	4
**LSHADE**	48.99	46.79	95.78	5
**LSHADE_SPACMA**	49.97	47.01	96.98	3
**UMOEA**	49.98	47.02	97	2
**WOA**	0.09	17.98	18.07	9
**AOA**	0.39	25.98	26.37	8
**CPSOGSA**	5.87	37.91	43.78	6

**Table 15:** Comparative results of Wilcoxon’s test for 10D, 30D, 50D and 100D benchmark functions.

Dimension	Algorithms	R^+^	R^-^	P-value	+	-	≈	Dec.
10	ADMO vs DMO	351.00	0.00	**0.000**	26	0	3	+
	ADMO vs LSHADEcnEpSin	50.50	4.50	**0.019**	8	2	19	≈
	ADMO vs LSHADE	55.00	0.00	**0.005**	10	0	19	≈
	ADMO vs LSHADE_SPACMA	43.50	1.50	**0.013**	8	1	20	≈
	ADMO vs UMOEA	28.00	0.00	**0.018**	7	0	22	≈
	ADMO vs WOA	435.00	0.00	**0.000**	29	0	0	+
	ADMO vs AOA	378.00	0.00	**0.000**	27	0	2	+
	ADMO vs CPSOGSA	276.00	0.00	**0.000**	23	0	6	+
30	ADMO vs DMO	425.00	10.00	0.103	28	1	0	+
	ADMO vs LSHADEcnEpSin	207.00	93.00	.050	15	9	5	≈
	ADMO vs LSHADE	187.00	66.00	**.000**	16	6	7	≈
	ADMO vs LSHADE_SPACMA	223.00	77.00	.037	15	9	5	≈
	ADMO vs UMOEA	165.00	111.00	.412	15	8	6	≈
	ADMO vs WOA	435.00	0.00	**0.000**	29	0	0	+
	ADMO vs AOA	435.00	0.00	**0.000**	29	0	0	+
	ADMO vs CPSOGSA	337.00	14.00	**0.000**	24	2	3	+
50	ADMO vs DMO	435.00	0.00	0.534	29	0	0	+
	ADMO vs LSHADEcnEpSin	200.00	151.00	0.585	13	13	3	≈
	ADMO vs LSHADE	197.00	154.00	**0.000**	14	12	3	≈
	ADMO vs LSHADE_SPACMA	258.00	120.00	0.097	15	12	2	≈
	ADMO vs UMOEA	146.00	179.00	.657	11	14	4	≈
	ADMO vs WOA	435.00	0.00	**0.000**	29	0	0	+
	ADMO vs AOA	435.00	0.00	**0.000**	29	0	0	+
	ADMO vs CPSOGSA	403.00	3.00	**0.000**	27	1	1	+
100	ADMO vs DMO	435.00	0.00	0.534	29	0	0	+
	ADMO vs LSHADEcnEpSin	200.00	151.00	0.585	14	14	1	≈
	ADMO vs LSHADE	197.00	154.00	**0.000**	14	12	3	≈
	ADMO vs LSHADE_SPACMA	258.00	120.00	0.097	15	11	3	≈
	ADMO vs UMOEA	146.00	179.00	.657	11	14	4	≈
	ADMO vs WOA	435.00	0.00	**0.000**	29	0	0	+
	ADMO vs AOA	435.00	0.00	**0.000**	29	0	0	+
	ADMO vs CPSOGSA	403.00	3.00	**0.000**	27	1	1	+

The LSHADE, LSHADEcnEpSin, LSHADE_SPACMA, and UMOEA came first in the different CEC competitions they entered. The performance of the proposed ADMO is compared with these algorithms and candidate representation of swarm-based (WOA, DMO) and physical-based (AOA, CPSOGSA) metaheuristic algorithms. It can be seen from the results that the proposed ADMO was very competitive with the high-performing algorithms (LSHADE, LSHADEcnEpSin, LSHADE_SPACMA, and UMOEA) across all dimensions considered. The DMO, AOA, and WOA performed poorly, failing to find optimal solutions for most benchmark problems, while the CPSOGSA performed relatively better, finding solutions for 3 functions in 10 dimensions. Generally, the performance of all the algorithms deteriorated significantly as the dimensions increased. However, the ADMO showed its stability and robustness by returning the best or most competitive solutions over all the dimensions considered.

**Fig 4:**
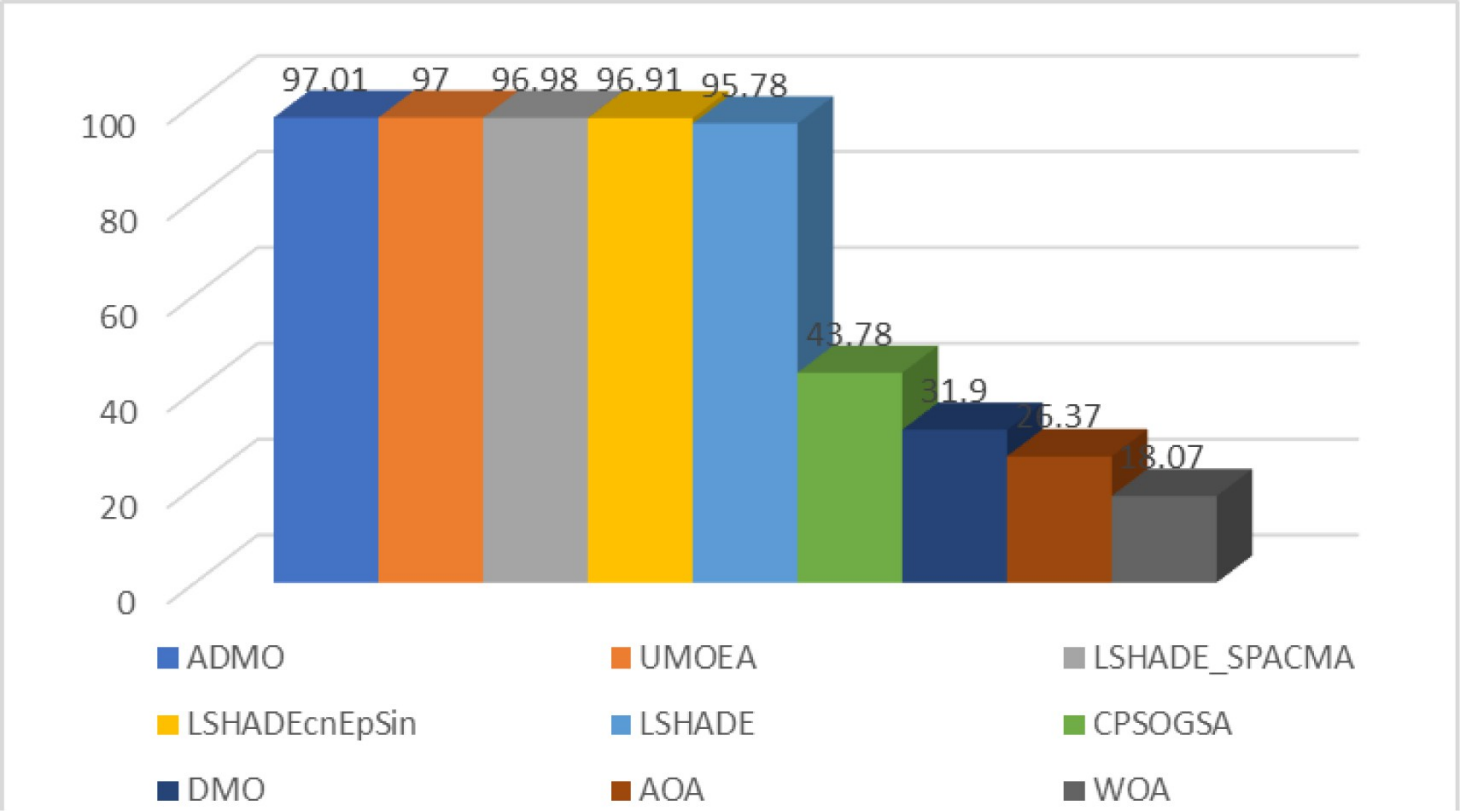
Graphical representation of the algorithm’s performance score.

The ranking of the algorithms considered based on the scoring system defined in [77]is presented in Table 14. Clearly, the five (5) high-performing algorithms were very competitive, with score differences ranging from 0.01 to 1.23, which is very small. Overall, the ADMO ranked first, outperforming the other algorithms in 10 and 30 dimensions, respectively. The graphical representation of the scores for each algorithm is shown in Fig 4.

The comparative results of all algorithms considered are tested statistically using Wilcoxon’s test, which is presented in Table 15. The results are presented for each dimension (10D, 30D, 50D, and 100D). From the results, the ADMO significantly outperforms the DMO, AOA, WOA, and CPSOGSA in all four (4) dimensions considered judging by the high R+ values returned by the ADMO. Also, the ADMO, UMOEA, LSHADE_SPACMA, LSHADEcnEpSin, and LSHADE were competitive, judging by the number of ties (≈) returned between their comparisons. At a significance level set at α = 0.05, the Wilcoxon’s test showed a significant difference in 16 out of 28 cases, which implies that the ADMO significantly outperformed 4 out of the 9 algorithms and insignificantly outperformed the remaining 4 algorithms.

In detail, the ADMO performed better, the same, less than the other algorithms considered in 138, 3, 91 out of 232 cases for 10 dimensions. In 30 dimensions, the ADMO performed better, the same, or less than the other algorithms in 171, 35, 26 out of 232 cases. Similarly, the ADMO performed better, the same, less than the other algorithms in 167, 52, 13 out of 232 cases for 50 dimensions. Finally, for 100 dimensions, the ADMO performed better, the same, less than the other algorithms in 168, 52, 12 out of 232 cases. Overall, the ADMO performed better, the same, less than the other algorithms in 644, 142, 142 out of 928 cases.

**Fig 5:**
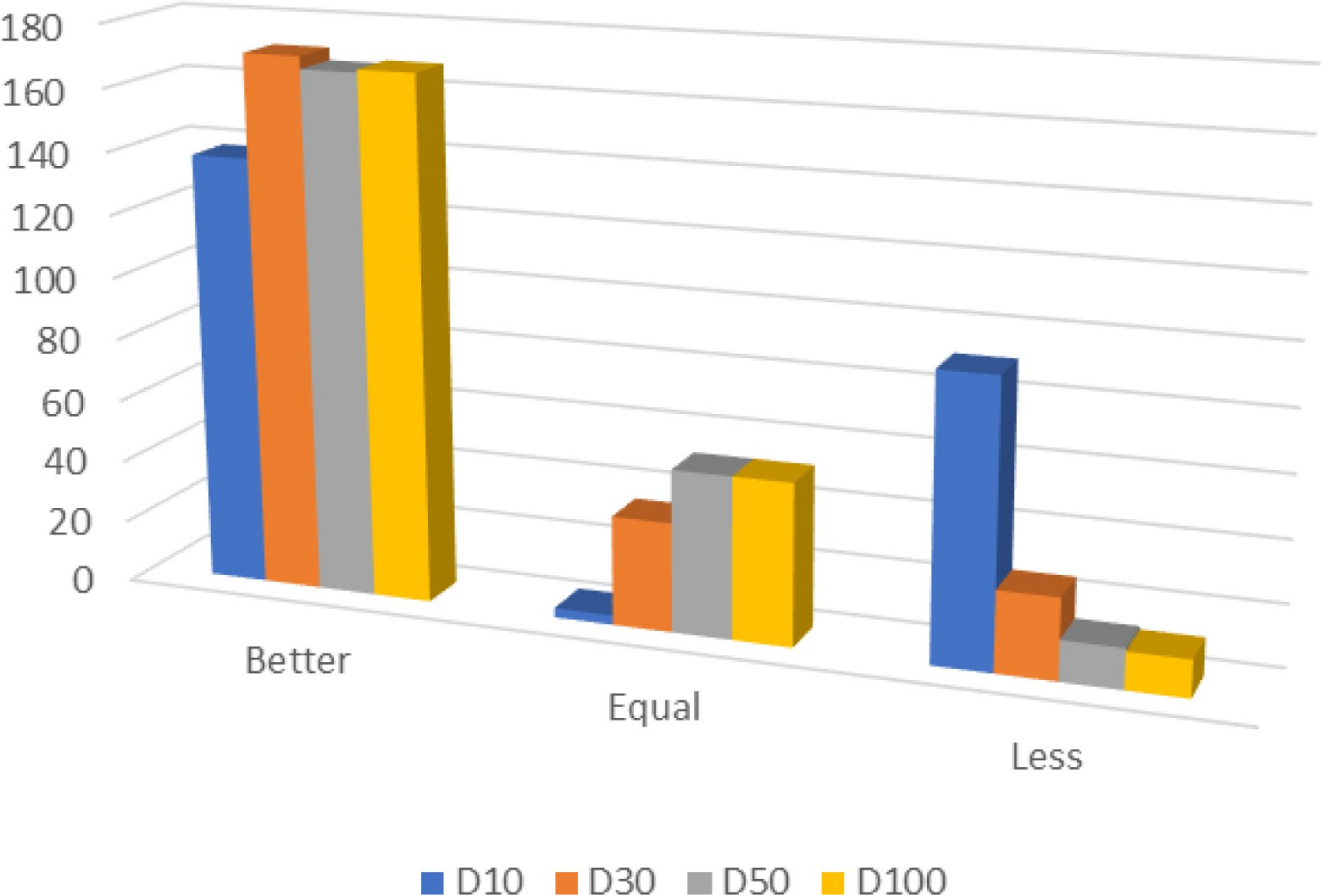
The comparative statistical result with growth in dimension.

Conclusively, the ADMO outperformed or was competitive in 85% of all cases. Also, Fig 5 shows the superiority of the proposed ADMO over the DMO and 7 other state-of-the-art algorithms considered across all the dimensions used in this study. The results also confirmed the searchability, stability, and efficiency of the ADMO in solving the optimization problems used in this study. The performance of ADMO was not hindered by the characteristics associated with the CEC 2017 problems, which are unimodal (separable and non-separable), multimodal (separable and non-separable), hybrid, and composite benchmark functions. This performance can be attributed to the balanced exploitation and exploration introduced by explicitly defining the predation, foraging and semi-nomadism, reproduction, and group splitting activities to carry out each optimization phase.

**Fig 6:**
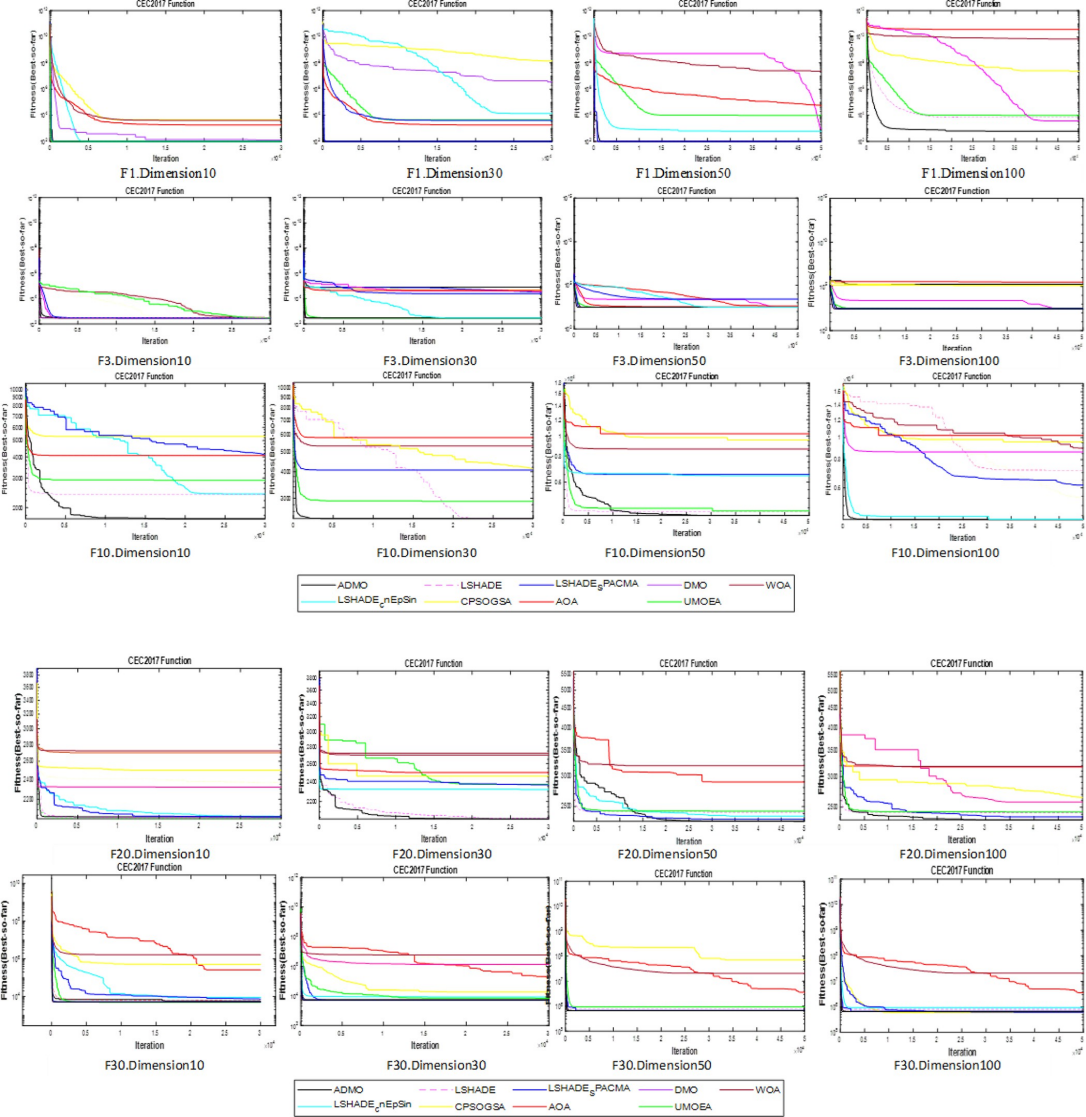
Convergence behavior of selected CEC 2017 functions.

Furthermore, the convergence behavior of all the algorithms considered and for all dimensions is shown in Fig 6. The ADMO showed a fast convergence speed early in the iteration process for all functions. This speed slows down in the middle, especially towards the end of the iteration process. Furthermore, the convergence figure of ADMO showed that global or near-global solutions are attained in a smaller number of iterations for most functions. The continuous exploitation and exploration further demonstrate the scalability of the ADMO until the stop criterium is met.

### 4.2. The CEC2011 real-world problems

#### 4.2.1. Result of ADMO for CEC 2011

The results of ADMO solving the CEC 2011 real-world problems are presented in Table 16. It should be noted that the value of the optimal solution to these problems is not available. However, the results are discussed based on four performance metrics (best, worst, mean, and standard deviation) used to summarize the results. The results are collated over 25 independent runs for all 22 benchmark functions. The population size and other algorithm-specific metrics remained as defined in Section 4.1. it can be observed that the ADMO consistently found the same solution over the 25 independent runs of the algorithm for F4, F8, and F10; this could be the optimal solution for these functions. For the rest of the function, the solution found was not consistent over the different runs of the algorithm, but they are very close to each other, judging by the very small deviation from the mean. A conclusion can be drawn that the ADMO is an effective tool for optimizing this set of problems. Next, the ADMO is compared with other algorithms to gauge its superiority and robustness further.

**Table 16:** Results of ADMO for CEC 2011.

Function	Best	Worst	Mean	Std
**F1**	0.00E+00	8.35E-02	4.56E-03	1.64E-02
**F2**	-2.84E+01	-2.76E+01	-2.84E+01	2.17E-01
**F3**	1.15E-05	1.15E-05	1.15E-05	6.98E-11
**F4**	0.00E+00	0.00E+00	0.00E+00	0.00E+00
**F5**	-3.68E+01	-3.49E+01	-3.58E+01	7.43E-01
**F6**	-2.92E+01	-1.86E+01	-2.60E+01	4.44E+00
**F7**	8.70E-01	1.30E+00	1.15E+00	1.08E-01
**F8**	2.20E+02	2.20E+02	2.20E+02	0.00E+00
**F9**	3.01E+05	3.01E+05	3.01E+05	1.88E+00
**F10**	-6.80E+00	-6.80E+00	-6.80E+00	0.00E+00
**F11**	5.11E+04	5.32E+04	5.24E+04	6.47E+02
**F12**	1.07E+06	1.08E+06	1.07E+06	1.44E+03
**F13**	1.54E+04	1.54E+04	1.54E+04	2.63E-06
**F14**	1.80E+04	1.82E+04	1.81E+04	3.99E+01
**F15**	3.27E+04	3.27E+04	3.27E+04	5.91E-01
**F16**	1.26E+05	1.29E+05	1.27E+05	9.39E+02
**F17**	1.87E+06	1.92E+06	1.90E+06	1.57E+04
**F18**	9.35E+05	9.44E+05	9.41E+05	3.01E+03
**F19**	9.42E+05	9.60E+05	9.48E+05	4.98E+03
**F20**	9.36E+05	9.47E+05	9.40E+05	3.27E+03
**F21**	1.26E+01	1.76E+01	1.51E+01	1.25E+00
**F22**	1.19E+01	1.30E+01	1.27E+01	1.48E+00

#### 4.2.2. Comparative results for CEC 2011

The comparative results of ADMO with other state-of-the-art algorithms used to solve the CEC 2011 real-world problems are presented in Table 17. The results are discussed based on the mean and standard deviation returned by the respective algorithms over 25 independent runs and the same experimental conditions as detailed earlier. The LSHADE, LSHADEcnEpSin, LSHADE_SPACMA, and UMOEA came first in the different CEC competitions they entered. The performance of the proposed ADMO is compared with these algorithms and candidate representation of swarm-based (WOA, DMO), human activity (gaining-sharing knowledge (GSK) based algorithm [60]), and physical-based (AOA, CPSOGSA) metaheuristic algorithms. It can be seen from the results that the proposed ADMO was very competitive with the high-performing algorithms (LSHADE, LSHADEcnEpSin, LSHADE_SPACMA, GSK, and UMOEA) across all 22 problems considered. The DMO, AOA, and WOA performed sub-optimally, failing to find optimal solutions for most benchmark problems except F4, while the CPSOGSA performed relatively better, closely following the six high performers.

**Table 17:** Comparative results for CEC 2011.

Algorithms	ADMO		LSHADEcnEpSin		LSHADE		DMO		LSHADE_SPACMA		UMOEA		WOA		AOA		CPSOGSA		GSK	
Function	Mean	Std	Mean	Std	Mean	Std	Mean	Std	Mean	Std	Mean	Std	Mean	Std	Mean	Std	Mean	Std	3.28E+00	5.21E+00
**F1**	4.56E-03	1.64E-02	1.10E+01	1.61E+00	1.61E+00	3.34E+00	1.76E+01	5.78E+00	1.12E+01	6.61E+00	6.22E-01	2.41E+00	1.93E+01	5.65E+00	2.50E+01	4.44E+00	1.81E+01	5.25E+00	- 1.13E+01	1.03E+00
**F2**	-2.84E+01	2.17E-01	-1.46E+01	2.33E+00	-2.60E+01	1.72E+00	-2.48E+01	1.84E+00	-2.83E+01	3.94E-01	-2.83E+01	3.31E-01	-2.51E+01	2.17E+00	-8.03E+00	1.37E+00	-3.82E+00	3.14E+00	1.15E-05	9.12E-13
**F3**	1.15E-05	6.98E-11	1.15E-05	8.90E-12	1.15E-05	8.75E-13	2.03E-01	1.23E-02	1.15E-05	6.78E-09	1.15E-05	9.07E-10	1.22E-01	2.45E-01	1.23E+00	1.09E+00	2.01E-02	1.09E-03	0.00E+00	0.00E+00
**F4**	0.00E+00	0.00E+00	0.00E+00	0.00E+00	0.00E+00	0.00E+00	0.00E+00	0.00E+00	0.00E+00	0.00E+00	0.00E+00	0.00E+00	0.00E+00	0.00E+00	0.00E+00	0.00E+00	0.00E+00	0.00E+00	- 2.06E+01	1.21E+00
**F5**	-3.58E+01	7.43E-01	1.00E+30	2.89E+14	-3.24E+01	1.22E+00	-2.39E+01	2.18E+00	-3.59E+01	6.69E-01	-3.57E+01	1.05E+00	-2.81E+01	3.46E+00	-2.10E+01	1.07E+00	-2.02E+01	7.38E+00	- 6.94E+00	2.48E+00
**F6**	-2.60E+01	4.44E+00	1.00E+30	2.89E+14	-2.63E+01	1.46E+00	-1.71E+01	2.36E+00	-2.91E+01	2.07E-01	-2.91E+01	1.64E-01	-1.91E+01	2.94E+00	-1.40E+01	1.22E+00	-1.24E+01	6.44E+00	1.78E+00	1.08E-01
**F7**	1.15E+00	1.08E-01	1.06E+00	7.92E-02	1.13E+00	1.54E-01	1.61E+00	1.73E-01	1.27E+00	8.82E-02	5.59E-01	9.95E-02	1.72E+00	1.97E-01	1.84E+00	7.80E-02	8.35E-01	1.81E-01	2.20E+02	0.00E+00
**F8**	2.20E+02	0.00E+00	2.20E+02	0.00E+00	2.20E+02	0.00E+00	2.22E+02	6.93E+00	2.51E+02	1.49E+01	2.20E+02	0.00E+00	2.56E+02	3.40E+01	2.85E+02	2.33E+01	3.02E+02	6.05E+01	2.11E+03	5.02E+02
**F9**	3.01E+05	1.88E+00	1.69E+05	1.01E+04	4.72E+05	8.86E+04	1.66E+06	7.50E+04	3.01E+05	3.02E+02	1.05E+06	1.66E+05	1.03E+06	4.73E+04	4.51E+06	2.26E+05	3.05E+05	9.21E+02	- 2.16E+01	1.19E-01
**F10**	-6.80E+00	0.00E+00	-2.17E+01	1.20E-01	-2.11E+01	2.01E-01	-1.12E+01	7.86E-01	-9.02E+00	5.24E+00	-1.11E+01	9.14E-01	-1.07E+01	7.80E-01	-1.09E+01	1.62E+00	-1.49E+01	2.30E+00	5.24E+04	6.88E+02
**F11**	5.24E+04	6.47E+02	5.21E+04	5.48E+02	9.23E+05	2.55E+05	3.97E+05	1.19E+05	8.55E+06	2.49E+05	3.19E+08	2.61E+07	1.27E+06	1.07E+05	6.42E+06	6.83E+04	9.84E+05	3.46E+05	1.07E+06	1.73E+03
**F12**	1.07E+06	1.44E+03	1.08E+06	9.44E+03	3.32E+06	6.23E+05	4.68E+06	4.60E+05	1.00E+30	1.48E+14	5.20E+06	3.71E+05	1.47E+07	7.88E+05	1.24E+06	1.07E+05	1.10E+06	3.15E+03	1.54E+04	2.44E+00
**F13**	1.54E+04	2.63E-06	1.54E+04	1.39E+00	1.54E+04	1.97E-01	1.55E+04	2.79E+01	1.00E+30	1.48E+14	1.55E+04	6.99E+00	1.56E+04	5.46E+01	1.56E+04	1.15E+02	1.55E+04	2.13E+01	1.84E+04	1.22E+02
**F14**	1.81E+04	3.99E+01	1.81E+04	3.37E+01	1.85E+04	3.71E+01	1.92E+04	2.58E+02	7.00E+29	4.83E+29	1.81E+04	4.52E+01	1.93E+04	2.12E+02	1.90E+04	1.52E+02	1.93E+04	1.86E+02	3.28E+04	1.55E+01
**F15**	3.27E+04	5.91E-01	3.28E+04	1.43E+01	3.28E+04	3.16E+01	3.32E+04	1.28E+02	1.00E+30	1.48E+14	5.13E+06	3.44E+06	4.06E+04	2.34E+04	3.37E+04	1.37E+03	3.31E+04	1.35E+02	1.35E+05	2.22E+03
**F16**	1.27E+05	9.39E+02	1.28E+05	8.99E+02	1.30E+05	7.07E+02	1.46E+05	7.97E+03	1.00E+30	1.48E+14	6.12E+07	1.51E+07	1.47E+05	6.99E+03	1.48E+05	3.58E+03	1.48E+05	3.82E+03	2.09E+06	1.20E+05
**F17**	1.90E+06	1.57E+04	1.90E+06	1.02E+04	1.92E+06	1.64E+04	1.56E+09	1.47E+09	1.00E+30	1.48E+14	1.86E+10	4.55E+09	1.01E+10	3.64E+09	1.40E+10	1.17E+09	2.18E+06	2.73E+05	1.27E+06	7.56E+04
**F18**	9.41E+05	3.01E+03	9.43E+05	3.27E+03	9.47E+05	3.68E+03	3.08E+06	9.94E+05	9.40E+05	1.15E+03	1.55E+08	2.01E+07	4.89E+06	5.36E+06	5.91E+07	8.67E+06	9.52E+05	5.86E+03	2.00E+06	1.36E+05
**F19**	9.48E+05	4.98E+03	9.45E+05	2.37E+03	1.22E+06	7.50E+04	4.24E+06	2.17E+06	9.44E+05	1.80E+03	1.50E+08	1.66E+07	6.57E+06	5.33E+06	5.94E+07	1.27E+07	1.39E+06	2.08E+05	1.29E+06	9.20E+04
**F20**	9.40E+05	3.27E+03	9.40E+05	2.31E+03	9.52E+05	9.28E+03	3.66E+06	1.75E+06	9.40E+05	2.05E+03	1.51E+08	1.38E+07	5.28E+06	3.00E+06	5.62E+07	1.01E+07	1.09E+06	3.50E+05	1.70E+01	3.11E+00
**F21**	1.51E+01	1.25E+00	1.51E+01	5.97E-01	1.43E+01	2.56E+00	1.00E+30	1.48E+14	4.06E+01	4.63E+00	4.55E+01	3.54E+00	2.58E+01	8.47E+00	1.81E+01	1.20E+00	2.37E+01	7.12E+00	1.29E+01	2.93E+00
**F22**	1.27E+01	1.48E+00	1.43E+01	2.13E+00	1.66E+01	1.15E+00	3.89E+01	8.73E+00	4.21E+01	3.71E+00	3.49E+01	3.17E+00	2.22E+01	3.16E+00	2.49E+01	2.43E+00	3.80E+01	6.50E+00	3.28E+00	5.21E+00

**Fig 7:**
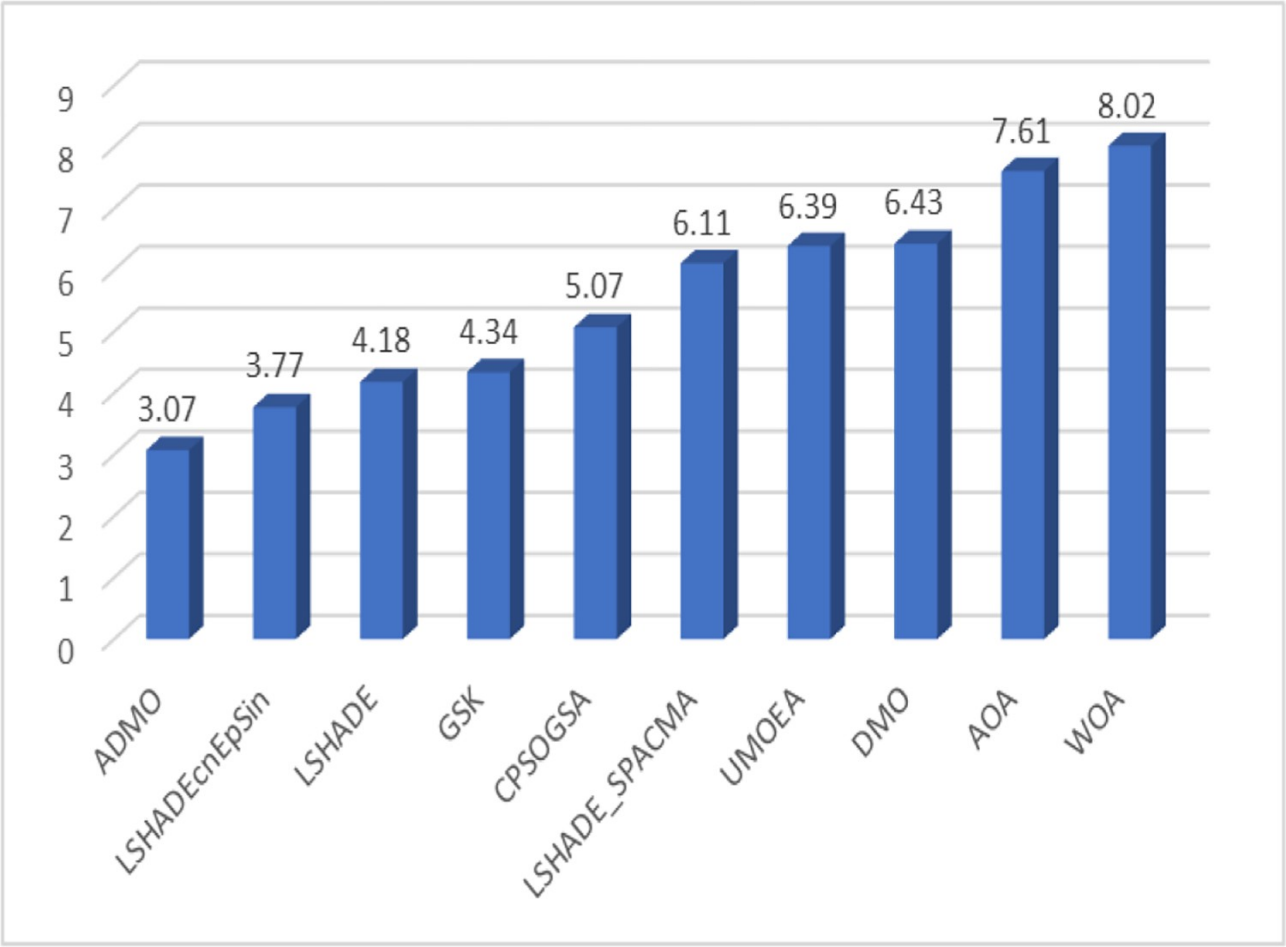
Graphical representation of algorithm’s performance ranking.

The ranking of the algorithms considered based on Friedman’s test is presented in Table 18. The implication is that the smaller the mean rank, the better the performance. The null hypothesis for Friedman’s test is that “there is no significant difference between the distributions of the obtained results.” At a significant tolerance level set at α=0.05, the test returned a p-value=0.000 which is less than α. Therefore, reject the hypothesis. Also, the ADMO returned the least mean rank and ranked first. Closely following ADMO is LSHADEcnEpSin, then LSHADE. The least three performing algorithms are the DMO, AOA, and WOA. The graphical representation of the performance ranking of the algorithms in CEC 2011 is shown in Fig 7.

**Table 18:** Friedman’s test results.

Algorithm	Mean Rank	Ranking
ADMO	3.07	1
LSHADEcnEpSin	3.77	2
LSHADE	4.18	3
GSK	4.34	4
CPSOGSA	5.07	5
LSHADE_SPACMA	6.11	6
UMOEA	6.39	7
DMO	6.43	8
AOA	7.61	9
WOA	8.02	10
Test Statistics^a^		
N	22	
Chi-Square	63.125	
df	9	
Asymp. Sig.	0.000	

A further statistical analysis was carried out using Wilcoxon’s test to show a pairwise performance comparison between ADMO and the remaining algorithms, and the results are summarized in Table 19. From the results, the ADMO significantly outperforms the UMOEA, LSHADE_SPACMA, LSHADE, DMO, AOA, WOA, and CPSOGSA in all 22 problems considered judging by the high R+ values returned by the ADMO. Also, the ADMO, LSHADEcnEpSin, and GSK were competitive, judging by the number of ties (≈) returned between their comparisons. At a significance level set at α = 0.05, the Wilcoxon’s test showed that the ADMO significantly outperformed 7 out of the 9 algorithms and insignificantly outperformed the remaining 2 algorithms. The results also confirmed the searchability, stability, and efficiency of the ADMO in solving the real-world optimization problems defined in CEC 2011 used in this study.

**Table 19:** Comparative results of Wilcoxon’s test for CEC 2011 real-world problems.

Algorithms	R^+^	R^-^	P-value	+	-	≈	Dec.
ADMO vs DMO	184.50	25.50	0.003	18	2	2	+
ADMO vs LSHADEcnEpSin	79.50	40.50	0.268	11	4	7	≈
ADMO vs LSHADE	155.00	35.00	0.016	15	4	3	+
ADMO vs LSHADE_SPACMA	112.50	23.50	0.021	13	3	6	+
ADMO vs UMOEA	102.00	18.00	0.017	12	3	7	≈
ADMO vs WOA	227.00	26.00	0.001	20	2	0	+
ADMO vs AOA	186.00	24.00	0.002	18	2	2	+
ADMO vs CPSOGSA	163.00	23.00	0.006	16	3	3	+
ADMO vs GSK	101.50	51.50	0.237	12	5	5	≈

**Fig 8:**
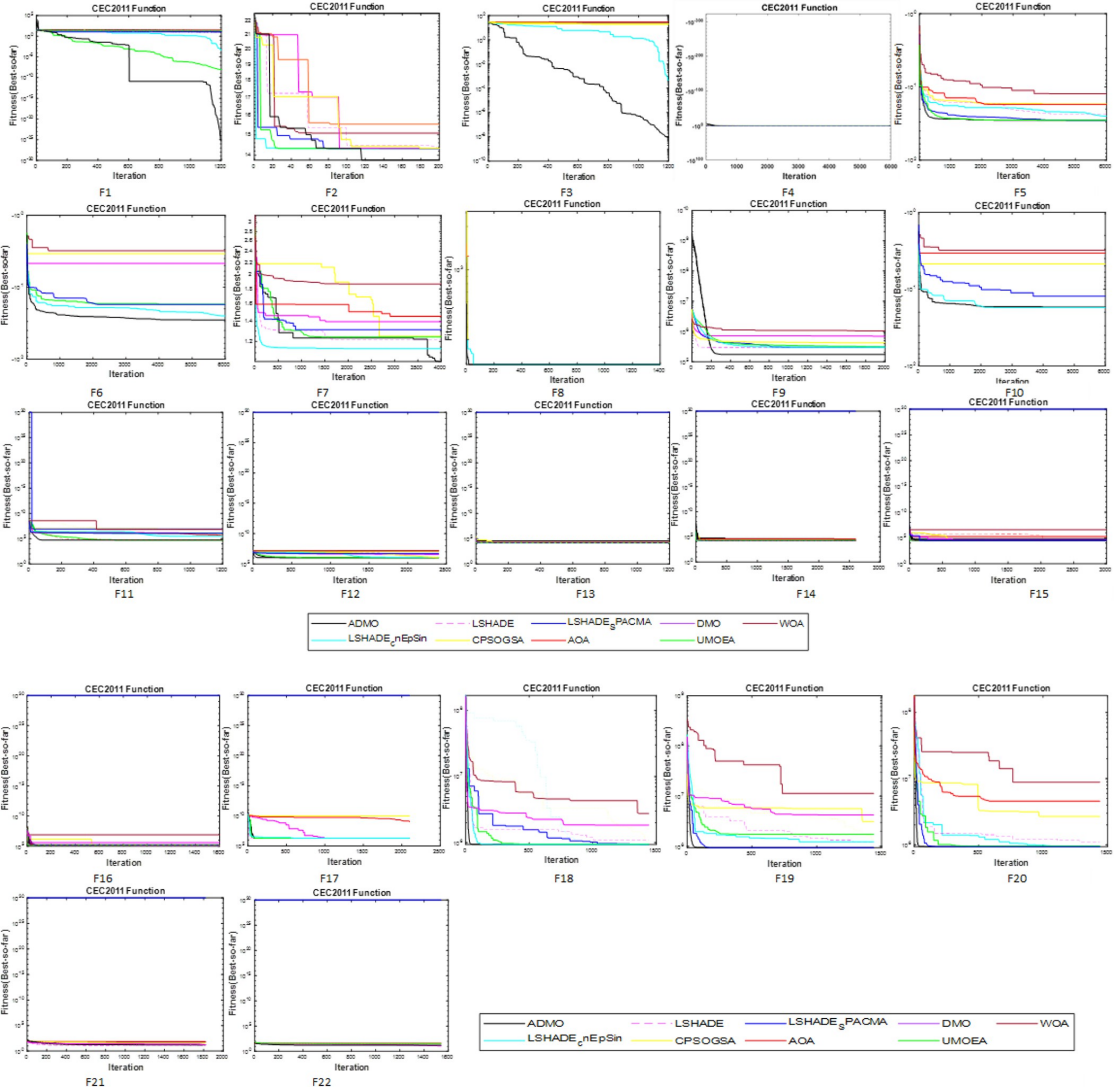
Convergence behavior for CEC 2011 real-world problems.

Furthermore, the convergence behavior of all the algorithms considered and for all 22 real-world problems is shown in Fig 8. The ADMO showed a fast convergence speed early in the iteration process for most functions except F1 and F3, which converged at the later stage of the iterations. This speed slows down in the middle, especially towards the end of the iteration process. Furthermore, the convergence figure of ADMO showed that global or near-global solutions are attained in a smaller number of iterations for most functions. The continuous exploitation and exploration further demonstrate the scalability of the ADMO until the stop criterium is met.

**Fig 9:**
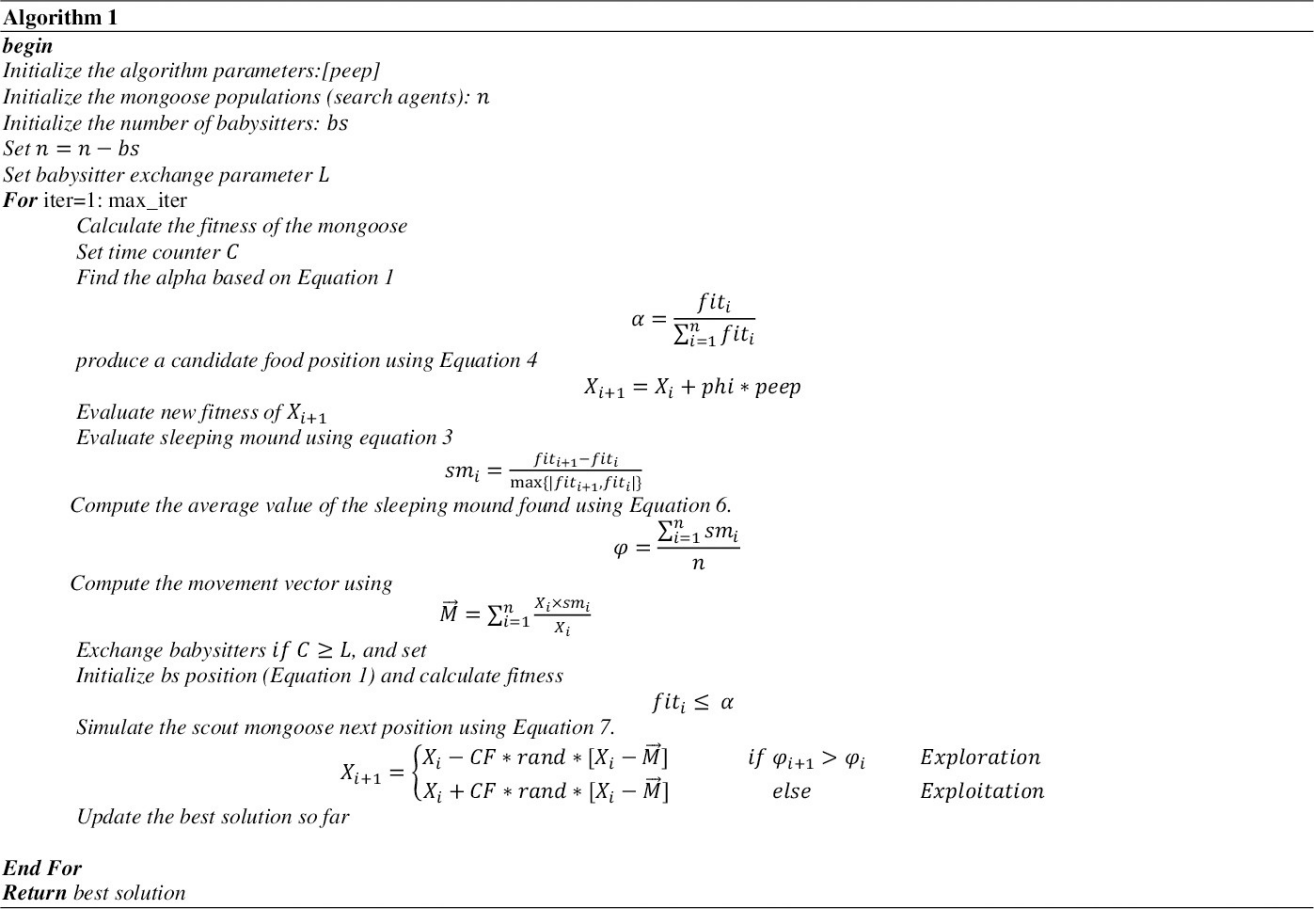
Algorithm 1.

**Fig 10:**
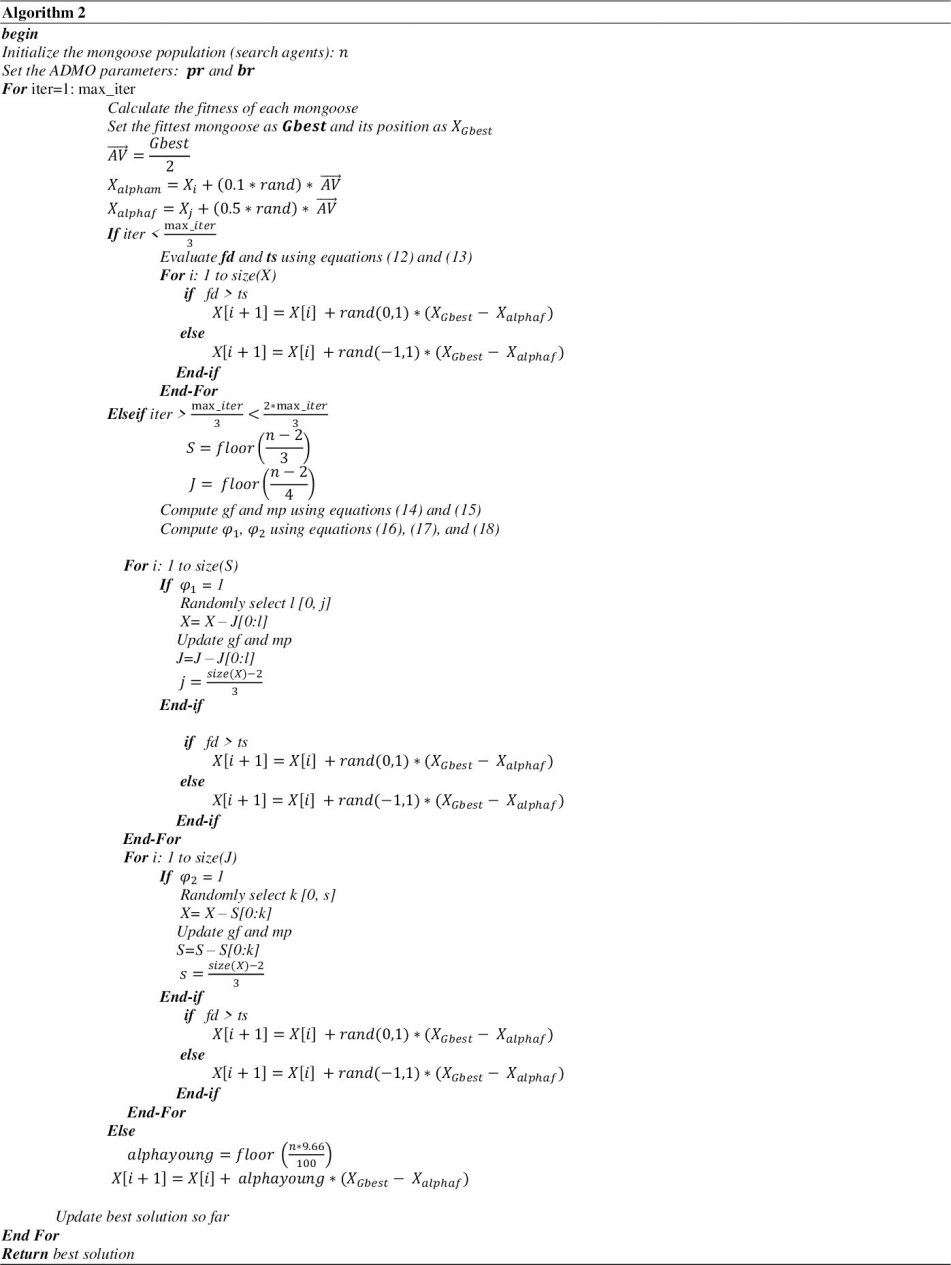
Algorithm 2.

### 4.3. Summary of results

To test the effectiveness and robustness of ADMO, it is applied to solve the CEC-2017 and CEC 2011 real-parameter benchmark and real-world optimization problems, respectively. Experimental results are compared with DMO and 7 other state-of-the-art algorithms, comprising 4 algorithms that came first in different CEC competitions (LSHADE, LSHADEcnEpSin, LSHADE_SPACMA, and UMOEA) and three other candidate representations of other categories of metaheuristic algorithms (AOA, GSK, CPSOGSA, WOA). The performance of the algorithms a scored using the metric defined in CEC 2017 technical report and Friedman’s test.

ADMO ranked first among all algorithms for CEC 2017, closely followed by UMOEA, LSHADE_SPACMA, and LSHADEcnEpSin. Furthermore, the obtained results were statistically analyzed using Wilcoxon’s test (a non-parametric test) with a significance level of 0.05. Again, the results confirmed the superiority and competitiveness of the ADMO with the compared algorithms for all functions in the test suite. The ADMO was further used to solve the set of real-world optimization problems proposed for the CEC2011 evolutionary algorithm competition. Generally, ADMO, LSHADE, LSHADEcnEpSin, LSHADE_SPACMA, GSK, and UMOEA performed significantly better than the DMO, AOA, CPSOGSA, and WOA on most functions.

The ADMO showed a fast convergence speed early in the iteration process for all functions for CEC 2017. Similarly, the ADMO also showed a fast convergence speed early in the iteration process for most functions in CEC 2011 except F1 and F3, which converged at the later stage of the iterations. This speed slows down in the middle, especially towards the end of the iteration process. Furthermore, the convergence figure of ADMO showed that global or near-global solutions are attained in a smaller number of iterations for most functions. The continuous exploitation and exploration further demonstrate the scalability of the ADMO until the stop criteria are met.

## 5. Conclusion and future work

The ADMO algorithm is an improvement of the newly developed DMO. It addresses the slow convergence due to alpha value and performs exploitation and exploration better than the original DMO. The ADMO incorporated four different social life structures of the dwarf mongoose to accomplish this. The predation and mound protection and the reproductive and group splitting behavior enhance the exploration and exploitation ability of the DMO. The ADMO also modifies the lifestyle of the alpha and subordinate group and the foraging and seminomadic behavior of the DMO. In the proposed ADMO, each candidate solution is represented by an individual dwarf mongoose in the entire population of dwarf mongooses. They cooperate as a group to carry out these different activities that have been mathematically modeled to enhance the optimization abilities of the DMO.

To test the effectiveness and robustness of the ADMO, it is applied to solve the CEC-2017 and CEC 2011 real-parameter benchmark and real-world optimization problems, respectively. Experimental results are compared with DMO and 7 other state-of-the-art algorithms, comprising 4 algorithms that came first in different CEC competitions (LSHADE, LSHADEcnEpSin, LSHADE_SPACMA, and UMOEA) and three other candidate representations of other categories of metaheuristic algorithms (AOA, GSK, CPSOGSA, WOA). The performance of the algorithms a scored using the metric defined in CEC 2017 technical report and Friedman’s test. The ADMO ranked first among all algorithms, closely followed by the 4 high-performing algorithms (LSHADE, LSHADEcnEpSin, LSHADE_SPACMA, and UMOEA). The DMO, AOA, and WOA performed poorly across all the optimization problems considered in this study.

The ADMO is easy to implement and has been proven reliable, efficient, and robust for real parameter optimization. The ADMO, as presented, is focused on solving the single constrained continuous optimization problem. However, in future work, efforts can be made to modify the ADMO to solve constrained multi-objective optimization problems, discrete optimization problems, practical engineering optimization problems, and a host of other real-world applications. Another exciting research direction is to look at ways individual dwarf mongooses can have unique parameters and evolving intelligence capabilities. Interestingly, future research studies may focus on applying the algorithm to solve high dimensions or large-scale global optimization problems. A complete parametric study of the ADMO is another useful prospective research direction. Finally, the ADMO may be hybridized with any other robust metaheuristic algorithm.

## Supporting information

S1 File Algorithms 1 and 2. (DOCX)
