# Supplementary material for: Advanced dwarf mongoose optimization for solving CEC 2011 and CEC 2017 benchmark problems
Source: PLoS One. 2022 Nov 2;17(11):e0275346. doi: 10.1371/journal.pone.0275346 (PMC9629639; doi:10.1371/journal.pone.0275346)
Supplement: S1 File — (DOCX) [file pone.0275346.s001.docx]

**Algorithm 1**

***begin***

*Initialize the algorithm parameters:[peep]*

*Initialize the mongoose populations (search agents):* $n$

*Initialize the number of babysitters:* $bs$

*Set* $n=n-bs$

*Set babysitter exchange parameter* $L$

***For*** iter=1: max_iter

*Calculate the fitness of the mongoose*

*Set time counter* $C$

*Find the alpha based on Equation 1*

$$\alpha=\frac{{fit}_{i}}{\sum_{i=1}^{n} {fit}_{i}}$$

*produce a candidate food position using Equation 4*

$$X_{i+1}=X_{i}+phi*peep$$

*Evaluate new fitness of* $X_{i+1}$

*Evaluate sleeping mound using equation 3*

${sm}_{i}=\frac{{fit}_{i+1}-{fit}_{i}}{max\{\left| {fit}_{i+1},{fit}_{i} \right|\}}$

*Compute the average value of the sleeping mound found using Equation 6.*

$$\varphi=\frac{\sum_{i=1}^{n} {sm}_{i}}{n}$$

*Compute the movement vector using*

$\vec{M}=\sum_{i=1}^{n} \frac{X_{i}\times{sm}_{i}}{X_{i}}$

*Exchange babysitters* $if C\geq L$*, and set*

*Initialize bs position (Equation 1) and calculate fitness*

$${fit}_{i}\leq\alpha$$

*Simulate the scout mongoose next position using Equation 7.*

$$X_{i+1}=\left\{ \begin{aligned} X_{i}-CF*rand*{[X}_{i}-\vec{M}] if \varphi_{i+1}>\varphi_{i} Exploration \\ X_{i}+CF*rand*{[X}_{i}-\vec{M}] else Exploitation \end{aligned} \right.$$

*Update the best solution so far*

***End For***

***Return*** *best solution*

**Algorithm 2**

***begin***

*Initialize the mongoose population (search agents):* $n$

*Set the ADMO parameters:* $\boldsymbol{pr}$ *and* $\boldsymbol{br}$

***For*** iter=1: max_iter

*Calculate the fitness of each mongoose*

*Set the fittest mongoose as* $\boldsymbol{Gbest}$ *and its position as* $X_{Gbest}$

$$\vec{AV}=\frac{Gbest}{2}$$

$X_{alpham}=X_{i}+\left( 0.1*rand \right)* \vec{AV}$

$$X_{alphaf}=X_{j}+\left( 0.5*rand \right)* \vec{AV}$$

***If*** *iter <* $\frac{max\_iter}{3}$

*Evaluate* ***fd*** *and* ***ts*** *using equations (12) and (13)*

***For*** *i: 1 to size(X)*

***if*** *fd > ts*

$X\left[ i+1 \right]=X\left[ i \right] +rand(0,1)*(X_{Gbest}- X_{alphaf})$

***else***

*X*$\left[ i+1 \right]=X\left[ i \right] +rand(-1,1)*(X_{Gbest}- X_{alphaf})$

***End-if***

***End-For***

***Elseif*** *iter >* $\frac{max\_iter}{3}<$ $\frac{2*max\_iter}{3}$

$$S=floor\left( \frac{n-2}{3} \right)$$

$$J= floor\left( \frac{n-2}{4} \right)$$

*Compute gf and mp using equations (14) and (15)*

*Compute* $\varphi_{1}$*,* $\varphi_{2}$ *using equations (16), (17), and (18)*

***For*** *i: 1 to size(S)*

***If*** $\varphi_{1}$ *= 1*

*Randomly select l [0, j]*

*X= X – J[0:l]*

*Update gf and mp*

*J=J – J[0:l]*

$j=\frac{size(X)-2}{3}$

***End-if***

***if*** *fd > ts*

$X\left[ i+1 \right]=X\left[ i \right] +rand(0,1)*(X_{Gbest}- X_{alphaf})$

***else***

*X*$\left[ i+1 \right]=X\left[ i \right] +rand(-1,1)*(X_{Gbest}- X_{alphaf})$

***End-if***

***End-For***

***For*** *i: 1 to size(J)*

***If*** $\varphi_{2}$ *= 1*

*Randomly select k [0, s]*

*X= X – S[0:k]*

*Update gf and mp*

*S=S – S[0:k]*

$s=\frac{size(X)-2}{3}$

***End-if***

***if*** *fd > ts*

$X\left[ i+1 \right]=X\left[ i \right] +rand(0,1)*(X_{Gbest}- X_{alphaf})$

***else***

*X*$\left[ i+1 \right]=X\left[ i \right] +rand(-1,1)*(X_{Gbest}- X_{alphaf})$

***End-if***

***End-For***

***Else***

$alphayoung=floor \left( \frac{n*9.66}{100} \right)$

$X\left[ i+1 \right]=X\left[ i \right]+ alphayoung*(X_{Gbest}- X_{alphaf})$

*Update best solution so far*

***End For***

***Return*** *best solution*
